# Mitochondrial hyperactivity and reactive oxygen species drive innate immunity to the yellow fever virus-17D live-attenuated vaccine

**DOI:** 10.1371/journal.ppat.1012561

**Published:** 2025-04-21

**Authors:** Samantha G. Muccilli, Benjamin Schwarz, Byron Shue, Forrest Jessop, Jeffrey G. Shannon, Charles L. Larson, Adam Hage, Seon-Hui Hong, Eric Bohrnsen, Thomas Hsu, Alison W. Ashbrook, Gail L. Sturdevant, Shelly J. Robertson, Joseph W. Guarnieri, Justin Lack, Douglas C. Wallace, Catharine M. Bosio, Margaret R. MacDonald, Charles M. Rice, Jonathan W. Yewdell, Sonja M. Best

**Affiliations:** 1 Innate Immunity and Pathogenesis Section, Laboratory of Neurological Infections and Immunity, Rocky Mountain Laboratories, NIAID, NIH, Hamilton, Montana, United States of America; 2 Cellular Biology Section, Laboratory of Viral Diseases, NIAID, NIH, Bethesda, Maryland, United States of America; 3 Research Technologies Branch, NIAID, NIH, Hamilton, Montana, United States of America; 4 Immunity to Pulmonary Pathogens Section, Laboratory of Bacteriology, NIAID, NIH, Hamilton, Montana, United States of America; 5 Laboratory of Virology and Infectious Disease, The Rockefeller University, New York, New York, United States of America; 6 Center for Mitochondrial and Epigenomic Medicine, Children’s Hospital of Philadelphia, Philadelphia, Pennsylvania, United States of America; 7 Integrated Data Sciences Section, Research Technologies Branch, NIAID, NIH, Bethesda, Maryland, United States of America; 8 Division on Human Genetics, Department of Pediatrics, Perelman School of Medicine, University of Pennsylvania, Philadelphia, Pennsylvania, United States of America; The University of Chicago, UNITED STATES OF AMERICA

## Abstract

The yellow fever virus 17D (YFV-17D) live attenuated vaccine is considered one of the most successful vaccines ever generated associated with high antiviral immunity, yet the signaling mechanisms that drive the response in infected cells are not understood. Here, we provide a molecular understanding of how metabolic stress and innate immune responses are linked to drive type I IFN expression in response to YFV-17D infection. Comparison of YFV-17D replication with its parental virus, YFV-Asibi, and a related dengue virus revealed that IFN expression requires RIG-I-Like Receptor signaling through MAVS, as expected. However, YFV-17D uniquely induces mitochondrial respiration and major metabolic perturbations, including hyperactivation of electron transport to fuel ATP synthase. Mitochondrial hyperactivity generates reactive oxygen species (ROS) including peroxynitrite, blocking of which abrogated MAVS oligomerization and IFN expression in non-immune cells without reducing YFV-17D replication. Scavenging ROS in YFV-17D-infected human dendritic cells increased cell viability yet globally prevented expression of IFN signaling pathways. Thus, adaptation of YFV-17D for high growth imparts mitochondrial hyperactivity to meet energy demands, resulting in generation of ROS as the critical messengers that convert a blunted IFN response into maximal activation of innate immunity essential for vaccine effectiveness.

## Introduction

Yellow fever virus (YFV) is the prototypical *Orthoflavivirus*, positive-stranded RNA arboviruses that cause significant global morbidity and mortality each year [1]. Between the 15^th^ and 19^th^ centuries, yellow fever was among the most devastating infectious diseases in old and new worlds, including a 1793 outbreak that killed approximately 10% of Philadelphia’s population in the USA [2–4]. Walter Reed discovered YFV as the etiological agent early in the 20^th^ century. In rapid succession the *Aedes aegypti* mosquito was identified as its vector and the YFV-17D strain was generated as one of the most effective vaccines of all time [5–7]. Despite this, YFV remains an important human pathogen, causing approximately 80,000–200,000 infections annually with an estimated 40% mortality rate, mainly in Central and South America, and Africa [8].

The YFV-17D vaccine was empirically derived by serial passage of a virulent YFV strain (YFV-Asibi) in mouse and chicken embryos [6,7]. Administered to more than 500 million people worldwide, YFV-17D elicits strong innate and adaptive immune responses, and confers lifelong immunity to yellow fever in more than 95% of vaccinees [9]. YFV-17D robustly induces cytotoxic T cells, T_H_1 and T_H_2 CD4 T cells, and neutralizing antibodies that persist for 40 or more years [10,11]. The strength and quality of the adaptive immune response is strongly influenced by detectable viremia [12] and a robust innate immune signature [13–15]. All three classes of interferons (IFNs) are linked to inducing and shaping the adaptive immune response to the virus, as well as controlling YFV-17D replication and dissemination *in vivo* [16–18]. While the vaccine is considered safe, a small number of vaccinees (1/250,000) develop yellow fever vaccine-associated viscerotropic disease or neurotropic disease [8,19]. Patients with compromised type I IFN function are at a higher risk of developing disseminating infection [20,21]. Therefore, the innate immune response is considered the cornerstone of the success of the YFV-17D vaccine in both control of virus replication and induction of adaptive immunity. The extent to which signaling pathways are uniquely engaged by YFV-17D to drive heightened IFN and inflammatory responses compared to a related orthoflavivirus, dengue virus (DENV), is central to our study.

Several different pattern recognition receptors (PRRs) can be activated in response to infection with orthoflaviviruses to initiate an antiviral response. These include RIG-I-Like Receptors (RLRs), toll-like receptors (TLRs) and the DNA sensing pathway cGAS-STING. Previous work examining IFN responses to YFV-17D used mouse bone marrow-derived dendritic cells (DCs) and suggested that multiple TLRs are engaged to produce IFNs and other pro-inflammatory cytokines [18]. However, YFV does not productively replicate in mouse cells including monocytes and DCs. This makes mice a poor model for understanding how innate responses are activated given that cytosolic RLRs are essential in initiating innate immune responses that control orthoflavivirus dissemination and tissue tropism, and license downstream adaptive immune responses in IFN-competent models [22].

RLRs signal through the adaptor protein mitochondrial antiviral signaling protein (MAVS) to induce expression of IFNs, chemokines and pro-inflammatory cytokines. MAVS is localized on mitochondria and peroxisomes, and its oligomerization on mitochondrial membranes is required for assembling the MAVS signalasome to activate IRF3, IRF7 and NFκB transcription factors [23]. Accordingly, viruses manipulate the structure and inter-organellar communication of the mitochondria, peroxisomes, and ER to dampen innate immune signaling and enhance replication. For example, DENV induces mitochondrial elongation and convoluted membrane formation resulting in reduced ER-mitochondria contact sites and dampened MAVS signaling [24]. Conversely, viral mitochondrial manipulation to meet the metabolic needs of viral replication and dampen MAVS-dependent signaling can result in the release of mitochondrial DNA sensed by cGAS-STING that activates the IFN response [25–27].

In addition to PRR signaling, metabolic dysregulation and cellular stress responses may contribute to immune responses to YFV-17D vaccination. In humans, symptomatic responses to YFV-17D vaccination correlated with ER stress, reduced tricarboxylic acid (TCA) cycle functionality, increased plasma levels of reactive oxygen species (ROS), and induction of cellular redox genes [28]. Furthermore, the magnitude of adaptive immune responses to vaccination can be predicted by gene expression involved in glycolysis and the integrated stress response [9,29]. Together, these findings indicate a role for metabolic stress in activating the immune response to YFV-17D vaccination. However, mechanisms of metabolic stress and how they integrate with pathogen sensing and innate immunity in the context of YFV-17D replication are not known. Here, we provide a molecular understanding of the linkage between metabolic stress and innate immune responses to the YFV-17D vaccine that underpin its resounding success.

## Results

### Hepatotropic orthoflaviviruses induce distinct type I interferon dynamics independently of mitochondrial morphology

**Fig 1:**
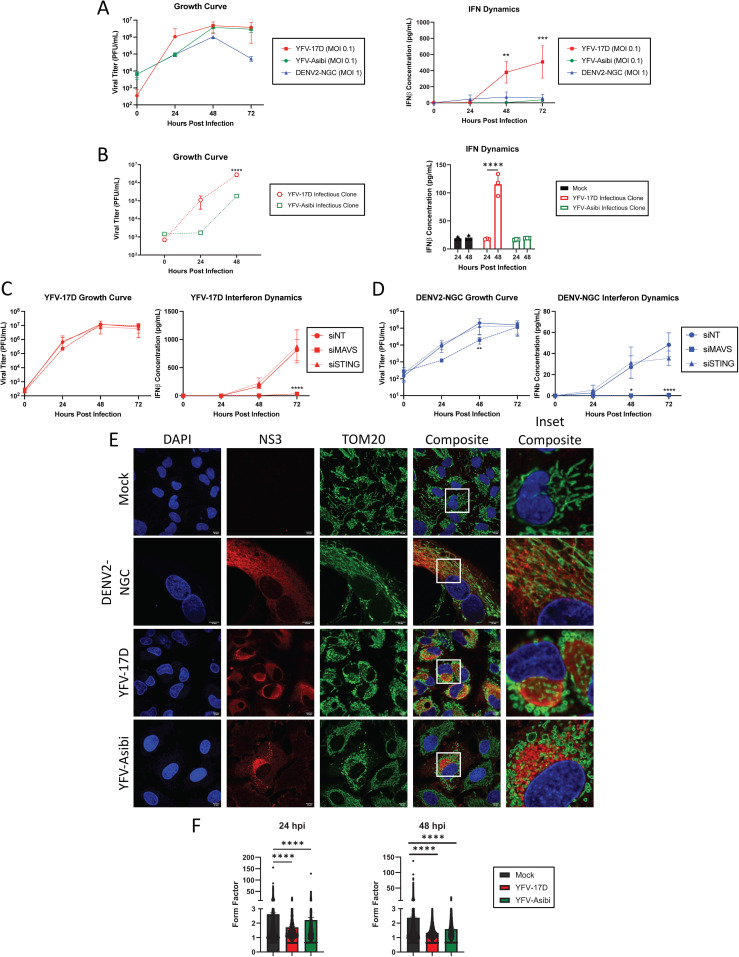
Dengue virus and yellow fever viruses modulate host mitochondrial and interferon dynamics differently. (A) Growth curve quantifying viral titer and ELISA quantification of IFNβ secreted by HepG2 cells infected with YFV-17D (MOI 0.1), YFV-Asibi (MOI 0.1), or DENV2 (MOI 1). (B) Growth curve quantifying viral titer and ELISA quantification of IFNβ secreted by HepG2 cells infected with molecular clone-derived YFV-17D (MOI 0.1) or YFV-Asibi (MOI 0.1). (C, D) Growth curve quantifying the viral titer and ELISA quantification of IFNβ secreted by HepG2 cells infected with C YFV-17D or D DENV2 and treated with siRNAs to knockdown MAVS, STING, or a non-targeting control. (E) Immunofluorescence (IF) of mitochondrial morphology following infection with mock, DENV2 (MOI 1), YFV-17D (MOI 0.1), or YFV-Asibi (MOI 1) for 48 h. Nuclei are stained with DAPI in blue, mitochondria are stained for TOM20 in green, and infected cells are stained for NS3 in red. (F) Quantification of the form factor of the mitochondrial network in HepG2 cells infected with mock, YFV-17D, or YFV-Asibi. Form factor was calculated using the following equation: Form Factor = (Perimeter)^2^/ 4π * Area. Values represent the mean ± SD (n = 3). Statistical significance was assessed using two-way ANOVA followed by Tukey’s (C and D) or Sidak’s (B) post hoc test for multiple groups or ordinary one-way ANOVA followed by Tukey’s post hoc test for multiple groups (F). *p < 0.05, **p < 0.01, ***p < 0.001, ****p < 0.0001.

To understand the contribution of mitochondrial dynamics to YFV-17D activation of innate immune responses, we first compared multi-cycle virus growth kinetics and type I IFN expression between YFV-17D, the parental YFV-Asibi, and DENV2 in HepG2 and Huh7 human hepatoma cell lines (Figs 1A and S1A). While the viruses attained similar peak titers in HepG2 cells, YFV-17D reached near peak titer in the first 24 hours, while YFV-Asibi and DENV2 required an additional 24 h. IFNβ was detected in YFV-17D-infected cell supernatants by 48 hpi, increasing for another day. Peak expression was at least 10-fold higher than present in YFV-Asibi or DENV2-infected cell supernatants, as previously reported [30]. Although the genome of YFV-17D is considered relatively stable [31], RNA-seq of our YFV-17D lab stock revealed a few consensus sequence mutations compared to the reference sequence (S1 Table). To alleviate concern that the major phenotypes that differentiate YFV-17D infection from its parental strain are a result of these mutations acquired during viral propagation, we also tested viruses derived from molecular clones. Recombinant YFV-17D and YFV-Asibi mirrored the tissue culture passaged lab stocks in growth kinetics and IFNβ secretion (Fig 1B). Of note, Huh7 cells failed to secrete measurable IFNβ following YFV-17D or DENV2 infection despite being highly permissible to infection (S1A Fig). Based on the robust IFN response, HepG2 cells were selected for further experiments. siRNA targeting critical adaptor proteins, MAVS and STING (S2A and S2B Fig), was used to characterize the dominant pathway of IFNβ induction. Depletion of MAVS resulted in a near complete reduction of IFNβ secretion following infection with YFV-17D (Fig 1C) or DENV2 (Fig 1D). Reduced STING expression did not affect IFNβ following YFV-17D infection, but reduced IFNβ production late in DENV2 infection, consistent with existing literature that mitochondrial DNA release late in DENV infection is sensed by cGAS/STING to contribute to induction of type I IFN [32]. These results confirm RLR-dependent signaling is a dominant mechanism of orthoflavivirus sensing in non-immune cells.

As orthoflaviviruses manipulate the structure and function of mitochondria, the predominant location of MAVS, we next examined the effects of viral infection on mitochondrial morphology via immunofluorescence staining for the outer membrane protein TOM20 and the viral nonstructural protein NS3 (Fig 1E). In mock-infected cells, mitochondria formed branched networks of intermediate length. As previously reported, DENV2 infection induced elongation and tangling of the mitochondrial network [24]. Conversely, infection with either YFV-17D or YFV-Asibi resulted in loss of branched mitochondrial networks in favor of individual, swollen mitochondria. Quantification of mitochondrial shape confirmed that YFV infection induced mitochondrial rounding at both 24hpi and 48hpi (Fig 1F). We extended these findings to Huh7 cells; DENV2 infection also induced mitochondrial elongation while YFV caused significant mitochondrial rounding and swelling (S1B Fig).

**Fig 2:**
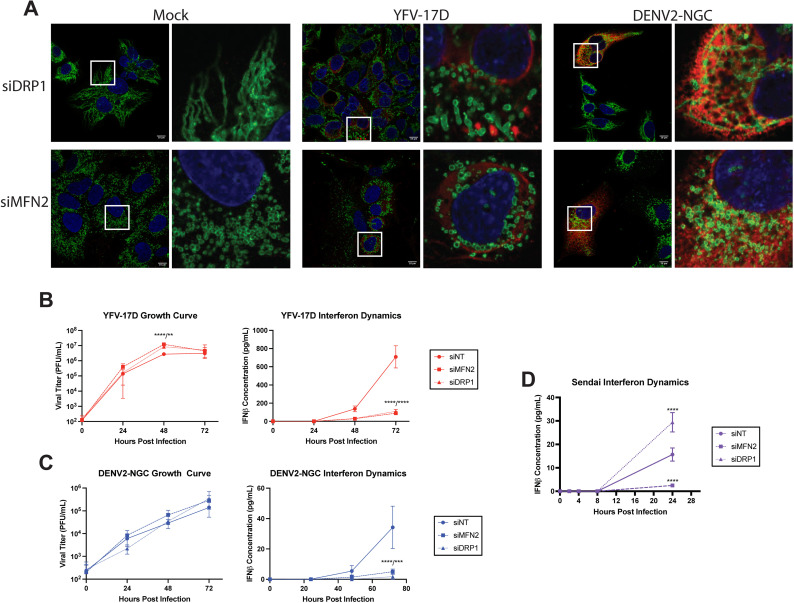
The type-I IFN response to YFV-17D and DENV2 is dissociated from mitochondrial morphology. **(A)** IF of mitochondrial morphology of HepG2 cells following infection with mock, YFV-17D (MOI 0.1), or DENV2 (MOI 1) and treated with siRNAs to knockdown DRP1 or MFN2 or nontargeting control. Nuclei are stained with DAPI in blue, mitochondria are stained for TOM20 in green, and infected cells are stained for NS3 in red. (**B and C)** Growth curve quantifying the viral titer and ELISA quantification of IFNβ secreted by HepG2 cells infected with **B** YFV-17D or **C** DENV2 and treated with siRNAs to knockdown MFN2, DRP1, or a non-targeting control. **(D)** ELISA quantification of IFNβ secreted by HepG2 cells infected with Sendai virus and treated with siRNAs to knockdown MFN2, DRP1, or a non-targeting control. Values represent the mean ± SD (n = 3). Statistical significance was assessed using two-way ANOVA followed by Tukey’s post hoc test for multiple groups **(B-C)**. *** p < 0.001 **** p < 0.0001.

Alterations in mitochondrial shape have a major impact on mitochondrial and cellular physiology. Mitochondrial elongation facilitates MAVS oligomerization and IFNβ production, while fission hinders MAVS’ ability to form high-order oligomers and impairs signaling [33,34]. We next explored how manipulating mitochondrial networks modulates orthoflavivirus induction of IFNβ using siRNA to deplete mitochondrial GTPases (S2C and S2D Fig). Dynamin-related protein 1 (DRP1) has a major role in mitochondrial fission, while mitofusin 2 (MFN2) mediates mitochondrial fusion [35]. As expected, DRP1 depletion elongated mitochondria while MFN2 depletion fragmented mitochondria in uninfected cells (Fig 2A). However, fragmentation of mitochondria occurred under conditions of DRP1 depletion in YFV-17D-infected cells, suggesting that viral infection supersedes DRP1 dependence for mitochondrial fission. By contrast, mitochondrial hyperfusion in DENV2-infected cells was dependent on MFN2, as reported [24]. Virus replication was slightly increased (YFV-17D; Fig 2B) or was not significantly affected (DENV2; Fig 2C) by loss of MFN2 or DRP1. Surprisingly, knockdown of either protein resulted in significant loss of IFNβ production following infection with either virus (Fig 2B and 2C). In contrast, in cells infected with Sendai virus (SeV, a paramyxovirus), a canonical inducer of MAVS-dependent IFNβ-expression, IFNβ secretion was increased in DRP1 siRNA-treated cells and decreased in cells depleted for MFN2 as has been previously published [33] (Fig 2D). Together with the high IFNβ response by YFV-17D infected cells that display a fragmented mitochondrial phenotype, these data indicate that MAVS-dependent expression of IFNβ in response to infection with these hepatotropic orthoflaviviruses is divorced from canonical mitochondrial morphology dynamics.

### YFV-17D drives rapid cellular energetic expenditure

**Fig 3:**
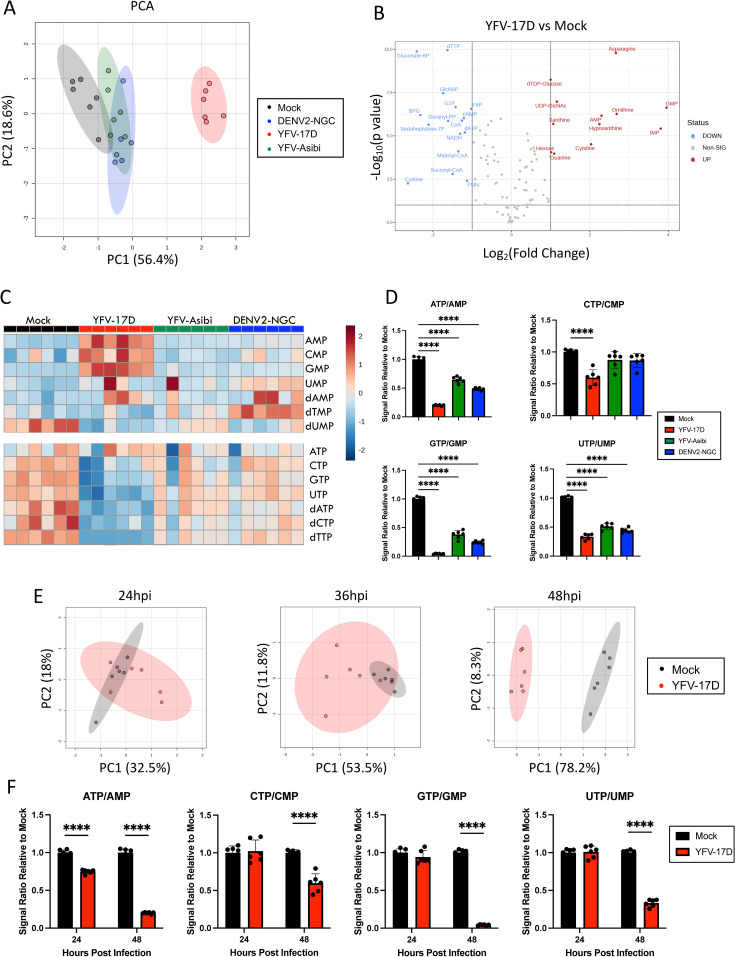
YFV-17D drives rapid cellular energetic expenditure. **(A)** Principle component analysis (PCA) of metabolomic differences between HepG2 cells infected with mock, DENV2 (MOI 1), YFV-17D (MOI 0.1), or YFV-Asibi (MOI 1) at 48hpi. **(B)** Volcano plots showing the significantly differentially abundant metabolites between YFV-17D infected HepG2 cells and mock-infected HepG2 cells at 48hpi as defined by metabolites with p < 0.1 equivalent to an FDR of 10% via a Benjamini Hochberg Procedure. Metabolites with a fold change of ≥ 2 or ≤ -2 are colored and labeled. **(C)** Metabolomic heatmap comparing the abundance of monophosphates and triphosphates following infection with mock, YFV-17D, YFV-Asibi, or DENV2. **(D)** Quantification of the triphosphate to monophosphate ratios in HepG2 cells infected with mock, YFV-17D, YFV-Asibi, and DENV2 at 48hpi. **(E)** PCA of metabolomic differences between mock and YFV-17D infected HepG2 cells at 24hpi, 36hpi, and 48hpi. **(F)** Quantification of the triphosphate to monophosphate ratios in mock and YFV-17D infected HepG2 cells at 24hpi and 48hpi. Values represent the mean ± SD (n = 6). Statistical significance was assessed using ordinary one-way ANOVA followed by Dunnett’s multiple comparisons test (D) or two-way ANOVA followed by Sidak’s multiple comparisons test **(F)**. **** p < 0.0001.

We next used a custom composite targeted liquid chromatography tandem mass spectrometry (LC-MS/MS) metabolomics method directed at central carbon, nitrogen, and oxygen metabolism to determine how orthoflavivirus-induced mitochondrial alterations correlate with metabolic changes in HepG2 cell cultures at 48 hpi. Principle component analysis (PCA) revealed that YFV-17D infection induced a distinct metabolic phenotype compared to mock, YFV-Asibi, or DENV2-infected cells (Fig 3A). Over 50% of measured metabolites were differentially abundant in YFV-17D infected cells compared to mock-infected cells with a FDR < 10% (Fig 3B). Conversely, YFV-Asibi and DENV2-infected cells had very few changes in metabolic intermediates relative to mock-infected cells (Fig 3A) despite major alterations in mitochondrial morphology. Analysis of differentially abundant metabolites revealed that YFV-17D infection starkly depleted high-energy triphosphates in favor of the low energy monophosphates (Fig 3C and 3D), indicative of either energetic exhaustion or a high rate of energetic expenditure. Triphosphate pools were reduced to a lesser extent in YFV-Asibi or DENV2 infected cells. Metabolomic analysis performed at 24 and 36 hpi revealed mock and YFV-17D infected cells were metabolically similar (Fig 3E). At these two timepoints, respectively, only two and five metabolites were differentially abundant between mock- and YFV-17D infected cells while triphosphate ratios were minimally different or unchanged (Figs 3F and S3). The tri/monophosphate ratios together with the lack of major metabolic changes indicate that this significant metabolic expenditure does not commence until after 36 hpi. Together, these data indicate that YFV-17D infection uniquely leads to a rapid and intense metabolic expenditure that coincides with IFNβ secretion.

### YFV-17D infection drives mitochondrial hyper-functionality and energetic overcommitment

**Fig 4:**
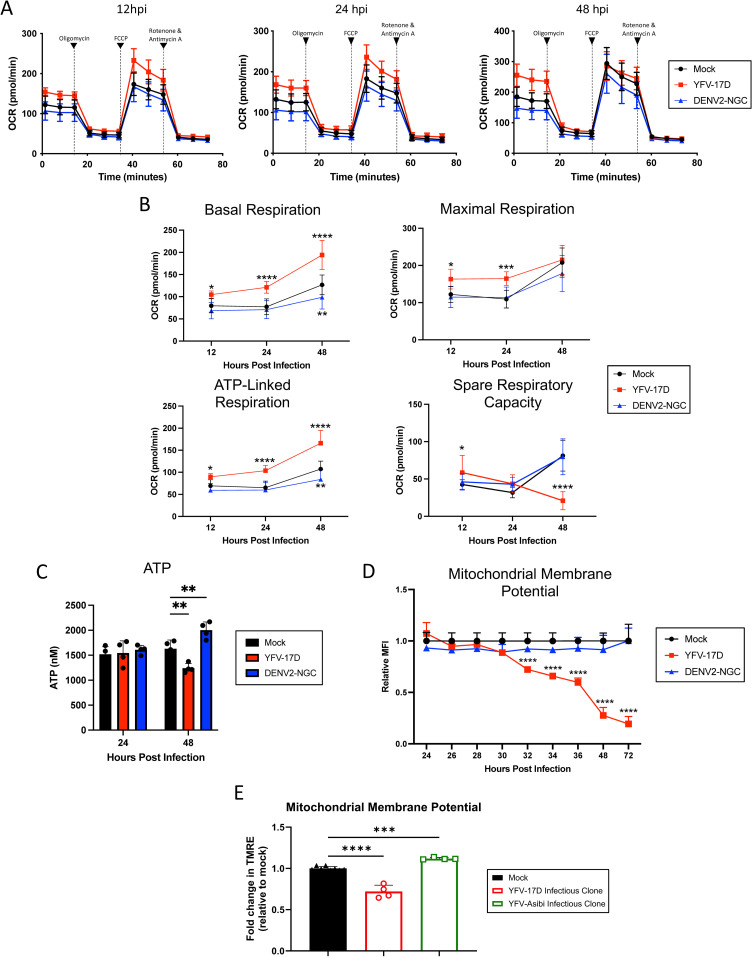
YFV-17D induces mitochondrial hyper-functionality and energetic overcommitment. **(A)** Seahorse XF cell mito stress test profile of HepG2 cells infected with mock, YFV-17D (MOI 0.1), or DENV2 (MOI 1) at 12hpi, 24hpi, and 48hpi. **(B)** Quantification of Seahorse XF mito stress test parameters (basal respiration, maximal respiration, ATP-linked respiration, and spare respiratory capacity) in mock, YFV-17D, or DENV2 infected HepG2 cells over 48 h. **(C)** Quantification of intracellular ATP levels in mock, YFV-17D, and DENV2 infected HepG2 cells at 24 hpi and 48 hpi. **(D)** Quantification of mitochondrial membrane potential (TMRE) in mock, YFV-17D, and DENV2 infected HepG2 cells between 24 hpi and 72 hpi. **(E)** Mitochondrial membrane potential at 48 hpi with molecular clone-derived YFV variants. Values represent the mean ± SD (B: n = 12, C: n = 4, D: n = 3, E: n = 4). Statistical significance was assessed using two-way ANOVA followed by Dunnett’s multiple comparisons test. * p < 0.05, ** p < 0.01, *** p < 0.001, **** p < 0.0001.

We next used seahorse extracellular flux analysis to measure oxygen consumption over time in the presence of electron transport chain (ETC) inhibitors to isolate the effects of infection on mitochondrial function [36]. First, basal respiration is measured, then ATP-synthase is blocked by oligomycin to calculate the proportion of oxygen used for ATP synthesis. Next, the proton gradient is collapsed by FCCP that allows electrons to flow unimpeded across the inner mitochondrial membrane to measure maximal respiration rate. Spare respiratory capacity, a measurement of the cell’s ability to respond to an increase in energetic demands during metabolic stress, can be calculated by subtracting the maximal respiration rate from the basal respiration rate. Finally, a combination of complex I inhibitor rotenone and complex III inhibitor Antimycin A entirely abolish mitochondrial respiration, enabling calculation of non-mitochondrial respiration (Fig 4A and 4B) [36]. We examined cells infected with YFV-17D and DENV2, but could not complete this for YFV-Asibi due to technical limitations at BSL-3.

Significantly increased basal respiration was evident as early as 12 hpi with YFV-17D (Fig 4B). Notably, by 48hpi, the basal respiration rate of YFV-17D infected cells was nearly equal to the maximal respiration rate. ATP-linked respiration is the primary contributor to this basal respiratory elevation in the YFV-17D infected cells as represented by the difference between basal respiration and respiration following oligomycin addition. Further, YFV-17D infected cells increased the rate of glycolysis between 24 and 48 hpi as measured by metabolomics and Seahorse assays (S4A–S4C Fig). Thus, YFV-17D infection maximizes mitochondrial respiration even under basal conditions, resulting in a significant decrease in mitochondrial spare respiratory capacity. In contrast, mitochondrial function was not changed by DENV2 infection, except for a decrease in basal respiration and glycolysis observed at 48 hpi.

In general, high mitochondrial respiration is associated with increased oxidative phosphorylation and ATP production [37]. Yet, the metabolomics analysis indicated that YFV-17D infection depletes ATP. Therefore, we sought to verify the metabolomics by quantifying total ATP concentration in infected cells. Indeed, despite substantial increases in total- and ATP synthase-linked respiration at 48 hpi, YFV-17D infected cells had a significantly lower standing pool of ATP compared to DENV2- or mock-infected cells (Fig 4C). The concomitant increases in glycolysis and ATP synthase-linked respiration, drop in spare respiratory capacity, maintenance of maximal respiration, and drop in triphosphate pools suggest extremely high energetic demands in the YFV-17D infected cells that is outpacing the maximally available triphosphate synthesis.

**Fig 5:**
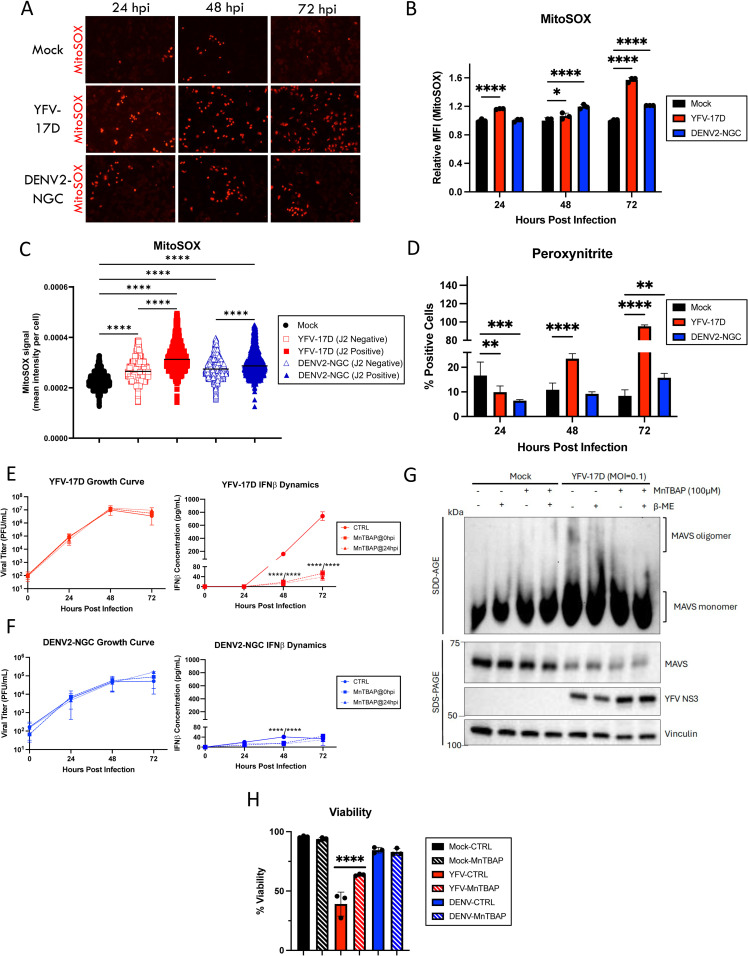
YFV-17D-induced ROS drives activation of the type I IFN response and cell death during YFV-17D infection. **(A)** Fluorescence indicating mitochondrial ROS production using MitoSOX dye in HepG2 cells infected with mock, YFV-17D (MOI 0.1), or DENV2 (MOI 1) at 24hpi, 48hpi, and 72hpi. **(B)** Quantification of MitoSOX staining in mock, YFV-17D, and DENV2 infected HepG2 cells using flow cytometry, or by **(C)** costaining with J2 mAb and quantitative microscopy to determine infection status of the cell. **(D)** Quantification of peroxynitrite staining in mock, YFV-17D (MOI 0.1), and DENV2 (MOI 1) infected HepG2 cells using flow cytometry. (**E and F)** Growth curve quantifying the viral titer and ELISA quantification of IFNβ secreted by HepG2 cells infected with **E** YFV-17D or **F** DENV2 and treated with MnTBAP [100 µ M] at 0hpi or 24hpi. **(G)** SDD-AGE analysis of MAVS oligomerization in mock- or YFV-17D-infected cells left untreated or treated with MnTBAP at time of infection. Duplicate samples were treated with beta-mercaptoethanol (β-ME) as a reducing control. One of two experiments shown. **(H)** Flow cytometric quantification of viability of mock, YFV-17D, and DENV2 infected HepG2 cells treated with MnTBAP or a vehicle control for 48 h. Values represent the mean ± SD (B, C, D, F: n = 3; E: n = 2). Statistical significance was assessed using two-way ANOVA followed by Tukey’s post hoc test for multiple groups (B, D) or ordinary one-way ANOVA followed by Tukey’s post hoc test for multiple groups (C, E, F, H). * p < 0.05, ** p < 0.01, *** p < 0.001, **** p < 0.0001.

While the seahorse analysis suggested increased mitochondrial function and health, the discordance of the flux data with ATP levels suggested that additional stressors and processes may be present in the mitochondria that were invisible to the measure of respiration under controlled conditions. To assess mitochondrial function in situ, we next measured mitochondrial membrane potential using flow cytometry. Mitochondrial membrane potential is driven by the ETC pumping protons into the intermembrane space that are then used by ATP synthase to produce ATP. In a healthy cell, at equilibrium, maintenance of membrane potential necessitates that the ETC is not rate limiting. Thus, at homeostasis it can be assumed that the rate of ETC turnover matches or exceeds the rate of ATP-synthase and other proton-gradient relaxation processes such as proton leak. Interestingly, we found mitochondrial membrane potential decreased precipitously in YFV-17D infected cells beginning at 32hpi, while DENV2-infected cells maintained mitochondrial membrane potential (Fig 4D). Loss of mitochondrial membrane potential at 48 hpi in YFV-17D-infected cells but not YFV-Asibi infected cells was confirmed using the viral molecular clones (Fig 5E). Finally, we tested two multiplicities of infection (MOIs) (S5A Fig). Induction of IFNβ secretion (S5B Fig) and loss of mitochondrial membrane potential was virus dose dependent for YFV-17D infection, but not for YFV-Asibi (S5C Fig). Intracellular genome copies of YFV-17D were 100 fold higher than YFV-Asibi at 24 and 48 hpi (S5C Fig). Thus, the phenotypes of mitochondrial damage and high IFNβ secretion represent virus-encoded differences between YFV-17D and YFV-Asibi associated with rapid genome replication.

Low mitochondrial membrane potential in conjunction with high ATP synthase-linked respiration indicates that mitochondrial depolarization is likely caused by ATP-synthase outpacing the ability of the ETC to replenish the proton pool in YFV-17D-infected cells. To our knowledge this phenomenon has not been directly observed in intact cells but previous studies have shown that isolated ATP-synthase responds directly to its substrates and products including ADP, inorganic phosphate, oxygen, and ATP [37–39]. Thus the described state where the ETC becomes rate limiting and ATP-synthase temporarily outpaces the ETC is possible but inherently unstable and places the cell under significant metabolic stress. The cell makes every attempt to meet this extreme metabolic burden including upregulating both glycolysis and mitochondrial respiration. Thus, mitochondria become hyper-functional and energetically overcommitted, upregulating ATP synthase activity to the point that ATP synthesis surpasses the rate of mitochondrial respiration. While minimal cell death was apparent at the time of these measurements up to 48 hpi, the cell is eventually no longer able to maintain the rapid rate of mitochondrial respiration and ATP synthesis and succumbs to cell death by 72 hpi as measured by Annexin V staining (S6 Fig).

### YFV-17D-induced ROS is responsible for activating type I interferon signaling

In addition to the generation of ATP, mitochondrial respiration also generates reactive oxygen species (ROS) that can activate a pro-inflammatory state in cells [40,41]. YFV-17D induced ROS over baseline levels measured by Mitosox labeling by 24 hpi whereas for DENV2, increased ROS required an additional 24 hours (Fig 5A and 5B). By 48 hpi, ROS staining was increased across the cell population compared to uninfected cells, but significantly elevated in viral dsRNA-positive cells suggesting infection directly drives ROS generation (Fig 5C). Treating cells with the cell permeable ROS scavenger MnTBAP at either 0 or 24 hpi did not impede replication of YFV-17D or DENV2. However, IFNβ production in YFV-17D infected cultures was reduced approximately 10-fold to levels equivalent to those produced following DENV2 infection whereas MnTBAP treatment only delayed IFNβ induction by DENV2-infected cells. (Fig 5E and 5F). A similar reduction in IFNβ secretion was observed in YFV-17D-infected cells treated with a second ROS scavenger, N-acetylcysteine (NAC) (S7A Fig).

Although mROS staining intensity was higher in cells infected with YFV-17D compared to DENV2 on a per cell basis, the magnitude of difference at 48 hpi does not seem sufficient to explain the significantly elevated IFNβ section in YFV-17D-infected cultures. MnTBAP also scavenges peroxynitrite, a ROS species generated from superoxide and nitric oxide [42]. Peroxynitrite induction closely mirrored that of IFNβ induction during YFV-17D replication (Fig 5D), and MnTBAP significantly decreased cellular peroxynitrite in infected cells (S7B Fig). Inhibiting nitric oxide synthase with L-NAME (S7C Fig) also significantly decreased IFNβ expression following infection, consistent with peroxynitrite being a major contributor to IFNβ secretion (S7C and S7D Fig). MnTBAP treatment also reduced MAVS oligomerization in YFV-17D-infected cells (Fig 5G), directly linking ROS production to its recognized function in MAVS oligomerization [43,44]. Finally, since high levels of ROS are cytotoxic, we investigated the effect of MnTBAP on cell viability. Treatment with MnTBAP partially rescued YFV-17D infected cells from death, consistent with linkage of type I IFN induction and cell death via ROS generation (Fig 5H). Taken together, our data indicate that YFV-17D uniquely induces maximal mitochondrial respiration that is unable to keep up with the metabolic needs of the cell. This metabolic stress results in enhanced ROS including peroxynitrite generation that amplifies MAVS oligomerization while also mediating cell death. Importantly, we also show that elevated IFNβ secretion is separable from virus replication and accompanying high levels of viral molecular patterns. Thus, ROS is identified as a critical driver of IFNβ expression by YFV-17D.

Given that the type I IFN response is incredibly energy intensive and that MAVS activation results in mitochondrial depolarization [45], we sought to determine if mitochondrial hyperactivity and energetic overcommitment was driven by a need to supply ATP for innate immune signaling or if this mitochondrial phenotype precedes IFN production. Inhibition of IFNβ production using either MnTBAP or siRNA to deplete cells of MAVS did not rescue the loss of mitochondrial membrane potential following YFV-17D infection (S8A and S8B Fig). Thus, mitochondrial overcommitment is driven directly by YFV-17D replication rather than the cell-intrinsic, energy intensive process of type I IFN signaling.

### ROS is required for human dendritic cells to mount an innate antiviral response to YFV-17D infection

**Fig 6:**
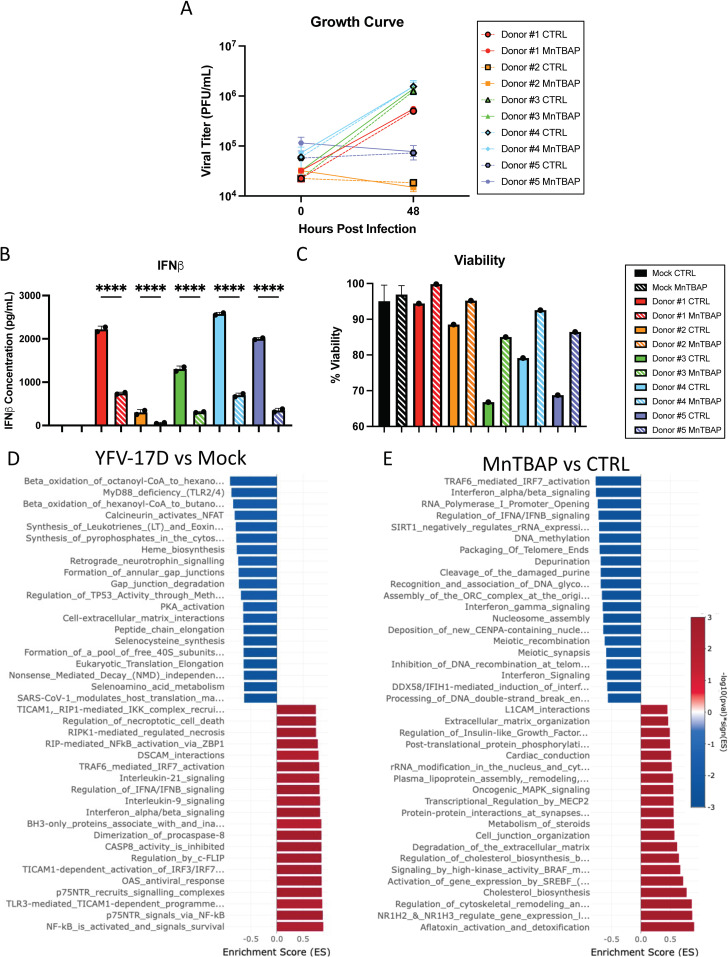
mROS is required for primary human dendritic cells to mount an innate immune response to YFV-17D infection. **(A)** Growth curve quantifying the viral titer of human DCs from five donors infected with YFV-17D (MOI 0.1) over a 48 h period. **(B)** ELISA quantification of IFNβ secreted by DCs and **(C)** cell viability following infection with YFV-17D and treatment with MnTBAP. **(D)** Pathway enrichment analysis of YFV-17D infected DCs compared to mock infected DCs at 48 hpi. **(E)** Pathway enrichment analysis of YFV-17D infected DCs treated with MnTBAP compared to vehicle control at 48hpi. Values represent the mean ± SD (n = 2). Statistical significance was assessed using ordinary one-way ANOVA followed by Sidak’s multiple comparisons test (B). **** p < 0.0001.

Dendritic cells (DCs) are professional antigen presenting cells that are both important targets of YFV infection and required for activation of naïve T cells. Given their critical role in vaccination of naïve individuals, we examined the role of ROS in virus-induced DC activation. Monocyte-derived DCs from five human donors were infected with YFV-17D in the presence of MnTBAP or vehicle control. While viral replication rates varied among donors (Fig 6A), IFNβ was detected in all infected cultures by 48 hpi (Fig 6B). As with HepG2 infection, MnTBAP treatment significantly decreased IFNβ expression and increased cell viability in all five donors without reducing viral replication (Fig 6A–6C).

Global gene expression changes were assessed in DCs by bulk RNA sequencing at 48 hpi (GEO accession number: GSE293880). Pathway enrichment analysis revealed that YFV-17D infection significantly upregulated mRNA involved in the innate immune response and cell death and downregulated protein translation associated mRNA (Fig 6D). MnTBAP treatment downregulated IFN signaling in infected cells (Fig 6E), with six of the top twenty downregulated pathways related to IFN signaling. Notably, multiple type I and III IFNs as well as interferon stimulated genes demonstrated ROS dependence (S2 Table). Global IFN signaling downregulation was accompanied by downregulation of genes involved in chemokine signaling, pointing to a broad role for ROS signaling in chemotaxis and DC based T cell help.

Consistent with a central role for oxidative stress responses in cell-intrinsic responses to infection, key antioxidant enzymes (*GSR*, *CAT*, *GPX1*, and *PRDX1*) were downregulated by YFV-17D and select genes (*SOD2*, *NFE2L2*, and *KEAP1*) were upregulated by MnTBAP treatment (S9A Fig). Thus, YFV-17D infection likely increases oxidative stress by inducing ROS in concert with reducing cellular antioxidant capabilities. Together, our findings demonstrate that expression of type I and III IFNs and chemokines, considered hallmarks of YFV-17D vaccine responses, depend on a virus-induced metabolic program culminating in high ROS generation coincident with initiation of RLR signaling. ROS is therefore the critical secondary messenger that converts relatively low-level PRR signaling into the extraordinary induction of innate immunity following YFV-17D infection.

## Discussion

Compared to other licensed vaccines, including live-attenuated viruses and recombinant viral vectors, the YFV-17D vaccine induces unique immune signatures in human vaccinees associated with detectable viremia and delayed but robust innate responses [46]. The magnitude of neutralizing antibody and cytotoxic T cell responses correlate with the viremic load and antiviral cytokine and chemokine responses [12,47]. In addition, cellular metabolic and stress responses enhance MHC I and II restricted antigen presentation and correlate with CD8 T cell responses [48,49]. YFV-17D vaccine gene signatures have been predominantly ascribed to TLR stimulation [9,18]. However, the dominant pattern recognition pathway controlling orthoflavivirus replication in IFN-competent animal models is MAVS-dependent [22]. Orthoflaviviruses also manipulate mitochondrial quality control pathways to facilitate replication and evade RLR signaling, amplifying the integrated stress and inflammatory responses [24,50]. These fundamental features of orthoflavivirus infection and innate immune activation prompted us to reexamine the contribution of MAVS-dependent signaling and mitochondrial function to YFV-17D triggering of innate immunity as measured by IFNβ secretion.

As shown previously, YFV-17D replicates rapidly when compared to its parental strain YFV-Asibi or DENV2 [30]. Rapid replication is associated with major metabolic and mitochondrial perturbations. YFV-17D infection maximizes mitochondrial respiration to a rate that is energetically unsustainable. Indeed, our data indicate that YFV-17D infection causes ATP-synthase to consume protons faster than they can be supplied by the ETC resulting in a rapid progressive reduction in mitochondrial membrane potential. Notably, this mitochondrial hyperactivity is independent of type I IFN signaling indicating that this is a process virally encoded rather than a side effect of an overactive innate immune response. Despite an extraordinary rate of ATP production as measured by highly elevated ATP-linked respiration, cells are still unable to meet the energetic demands of infection resulting in depletion of standing ATP pools. The onset of this metabolic stress is coincident with ROS generation and RLR activation. Thus, the metabolic stress of YFV-17D infection drives a reprioritization of nutrient/energetic resources to ROS generation and cytokine production prior to cell death. Importantly, blocking ROS-based signaling did not reduce viral replication. Thus, even in the presence of high levels of viral molecular patterns, ROS is the key factor that translates relatively low RLR pathway activation into the YFV-17D hallmark innate immune signature. In human DCs, ROS signaling was critical for expression of type I and III IFNs, a spectrum of chemokines, ISGs, and cell death pathway activation, all of which likely contribute to an adaptive immune response that confers lifelong protection from yellow fever. Importantly, this study reveals the cell biology of infection contributing to gene signatures associated with symptomatic responses and correlated with the magnitude of adaptive immunity in humans following YFV-17D vaccination [9,13–15,29].

Mitochondrial fusion, fission, and mitophagy are essential for maintaining cellular function. Viruses manipulate mitochondria to enhance replication through provision of metabolic intermediates for RNA replication as well as suppression of innate immune responses and apoptosis [51–53]. During RLR signaling, cells with defective autophagy accumulate ROS, and ROS accumulation can activate RLR signaling in autophagy-deficient cells [54]. Viral inhibition of mitophagy, and autophagy more generally [55], could contribute to the phenotypes demonstrated here given that the related Zika virus antagonizes mitophagy [50], and that the loss of mitochondrial membrane potential and mitochondrial fragmentation should trigger a mitophagy response. In contrast to that observed in YFV-17D-infected cells, MAVS activation is aided by an intact mitochondrial membrane potential, and increased mitochondria surface area and ER-contacts [34,52,56]. Indeed, we confirmed important aspects of this model using Sendai virus as a canonical RLR signaling activator. In Sendai virus infected cells, depleting MFN2 fragments mitochondria and reduces IFNβ secretion while depleting DRP1 elongates mitochondria and increases IFNβ secretion. However, while both YFV-Asibi and YFV-17D induced mitochondrial fragmentation, mitochondria in YFV-17D infected cells support high MAVS-dependent IFNβ secretion. We hypothesize that wild type YFV evolved to fragment mitochondria to dampen MAVS signaling to enhance replication and that this property was maintained in generating YFV-17D [57]. However, in the case of YFV-17D, infection-induced metabolic and oxidative stress negates any advantage provided by fragmented mitochondrial morphology. While YFV-induced mitochondrial fragmentation is independent of DRP1 (the major GTPase responsible for fission in uninfected cells), YFV-17D-induced IFNβ required both DRP1 and MFN2. MFN2-dependence is consistent with its role in adapting macrophage mitochondrial respiration to metabolic stress and promoting mROS generation [58]. The requirement for DRP1 in YFV-induced MAVS-dependent signaling is puzzling, particularly as DRP1 is inactivated by TBK1 to facilitate MAVS responses [59], necessitating further work to interpret this finding. Nevertheless, our results indicate that while mitochondrial morphology strongly contributes to MAVS-based anti-viral immunity, other factors can override its canonical role.

Viral-induced alterations in metabolism favoring glycolysis, as we observed in YFV-17D-infected HepG2 cells, likely evolve in part to increase nucleotide levels via the pentose phosphate pathway to support viral replication [60]. Similar metabolic reprogramming is critical in allowing virus-activated DCs to become optimal antigen-presenting cells. Increased glycolytic flux is required for TBK1-, IKKε- and Akt- dependent DC activation [61]. MAVS signaling is also regulated by glycolysis and activates the same kinases, consistent with these processes being linked in DCs [62]. Immune cell activation is also closely associated with ROS production. Similarly, mitochondrial hijacking by DENV, influenza A, and vesicular stomatitis virus results in ROS generation that enhances RLR and MAVS signaling [63,64]. We confirmed a requirement for ROS in early expression of IFNβ by DENV-infected cells. While ROS is generally involved in RLR innate immune activation, the timing and magnitude of ROS-dependent IFNβ secretion potentially associated with generation of peroxinitrite points to critical differences between YFV-17D and related viruses.

Although the precise mechanism linking ROS to MAVS-dependent RLR signaling remains to be elucidated in the context of YFV-17D, several possibilities exist. Activated MAVS forms large, prion-like surface mitochondrial aggregates that are necessary and sufficient to activate downstream type I IFN signaling. It is well established that ROS-based MAVS aggregation generated by virus infection, chemicals, or systemic lupus erythematosus facilitates type I IFN signaling [43]. It is hypothesized that ROS oligomerizes MAVS by oxidizing specific cysteine residues, promoting disulfide bond formation and/or by peroxidizing lipids in the mitochondrial membrane, favoring MAVS transmembrane domain oligomerization [43,44]. Indeed, we observed that MAVS oligomerization was dependent on ROS in YFV-17D-infected cells. Thus, ROS acts at, or apical to, MAVS activation in this context. However, we have not ruled out that ROS can participate at additional points in the pathway, including activation of NF-κB, AP-1, MAPK, and PI3K pathways [65]. Due to its relative magnitude and timing of induction, we hypothesize that peroxinitrite is the specific ROS driving the response, which via various alterations to proteins, lipids, sugars and nucleotides [66,67], can independently influence metabolism and immune responses [68–70]. Thus, additional studies are required to precisely define the contribution of ROS-based mechanisms to YFV-17D immune activation in liver cells and DCs.

Much also remains to be learned about whether our findings relate to YFV-17D attenuation. Our data indicate that high metabolic burden is critical in augmenting the innate immune response to YFV-17D relative to YFV-Asibi and DENV2, which may be driven by rapid genome replication. Due to technical limitations in our BSL3, we did not directly assess ROS generation or Seahorse assays in YFV-Asibi-infected cells. However, the observations that mitochondrial membrane potential remains intact, that ATP levels are not overly diminished beyond that for DENV2-infected cells, and the metabolomic profiles were also similar to DENV all suggest that YFV-Asibi-infected cells are meeting energetic demands which requires functional mitochondria. YFV-17D differs from YFV-Asibi at 68 nucleotide positions, resulting in 32 non-synonymous codon changes scattered throughout the genome. Twelve changes are present in the viral envelope protein [71], conferring increased viral attachment and entry, increasing replication and enhancing proinflammatory pathway activation [30,72]. This may contribute to our finding that robust YFV-17D replication leads to metabolic stress and mitochondrial overcommitment, culminating in ROS-induced type-I IFN signaling and cell death. In this case, paradoxically, high viral replication would be beneficial to the host as it leads to recognition and control of YFV-17D before significant viral dissemination can occur. Substitutions in non-structural proteins may also contribute to high RNA replication, or directly interfere with mitochondrial function. Indeed, mitochondrial manipulation has been attributed to nonstructural proteins NS4A and NS5 of DENV and ZIKV, respectively [24,50]. Future reverse genetic-based mapping studies using the molecular clones used herein should elucidate the relevant mutations in YFV-17D responsible for its remarkable vaccine efficacy, and could be used to further examine how metabolic control in antigen presenting cells affects long-term immunological memory [48,49,62,73]. This information will be useful for designing not only future orthoflavivirus vaccines, but likely vaccines for a wide variety of viruses.

## Experimental procedures

### Cell culture

We cultured HepG2 cells (ATCC) and Vero E76 cells (ECACC) in DMEM (Gibco) supplemented with 10% FBS (Cytiva) and 1% penicillin/streptomycin (Gibco) at 37˚C and 5% CO_2_. We cultured C636 mosquito cells (ATCC) in MEM (Gibco) supplemented with 10% heat-inactivated FBS, 2mM Glutamine (Gibco), 1% NEAA (Gibco), and 1% penicillin/streptomycin at 32˚C and 5% CO_2_.

### Virus infections

We used the following viruses in this study: yellow fever virus (YFV) strain 17D (from NIH Biodefense and Emerging Infections Research Recourses Repository, NIAID, NIH, NR116); YFV strain Asibi (from University of Texas Medical Branch World Reference Center for Emerging Viruses and Arboviruses); dengue virus (DENV) strain New Guinea C (from Dr. Adolfo García-Sastre); Sendai virus (SeV) strain Cantell (from Charles River Laboratories). We performed all procedures with YFV-Asibi under biosafety level-3 (BSL-3) conditions at the Rocky Mountain Laboratories Integrated Research Facility (Hamilton, MT). We propagated orthoflavivirus working stocks on C636 cells for 2 passages and titrated by plaque assay on Vero cells. Multiplicity of infection (MOI) is represented as plaque forming units (PFU) per cell. For all experiments, we infected cells for 1 h at 37˚C to enable virus adsorption before removing virus inoculum and replacing with fresh culture medium. Sampling immediately after the 1 h virus adsorption is represented as the 0 hpi timepoint.

### Immunofluorescence

4 well Lab-Tek II chamber coverglass slides (Thermo Fisher Scientific) were coated with poly-l-lysine for 1 h at 37˚C prior to seeding cells. Following the culture period, cells were washed and fixed cells for 15 min at 37˚C in 4% paraformaldehyde (Electron Microscopy Sciences) diluted in DMEM. Following fixation, cells were washed twice and permeabilized with 0.1% Triton X-100/sodium citrate (Sigma Aldrich) for 15 min at room temperature and blocked in 3% normal goat serum for 1 h. Slides were incubated overnight at 4˚C in primary antibody diluted in 1% normal goat serum (Abcam), washed, and incubated with secondary antibody diluted in 1% normal goat serum for 1 h at room temperature followed by Hoescht (Thermo Fisher Scientific) for 5 min at room temperature. Slides were imaged using either a Zeiss LSM710 confocal microscope or a Leica Stellaris 8 confocal microscope and images analyzed using the FIJI software.

### Mitochondrial form factor

FIJI software was used to analyze the mitochondrial shape in Fig 1E. In each image, we identified regions of interest as infected cells based on positive staining for NS3. We then utilized a series of filters to segment and skeletonize the mitochondria network. Briefly, for each image, we applied an unsharp mask, enhanced local contrast, and applied a median filter to clearly visualize the mitochondrial network. We then used the “tubeness” and “skeletonize” tools to convert the mitochondrial network into discreet units for analysis. We used the “analyze particles” and “analyze skeleton” tools to obtain various features of the mitochondria including perimeter and area which were later used to determine the form factor. The number of mitochondria analyzed was not normalized across experimental groups as it was dependent both on the number of infected cells in each field of view and varied depending on mitochondrial morphology (networked vs fragmented). However, at least 2000 mitochondria were analyzed in each experimental group. For each experimental group, we analyzed at least 10 fields of view across 2–4 independent experiments.

### Fluorescence detection of ROS

HepG2 cells (3 x 10^4^ per well) in 24-well μ-Plates (Ibidi) coated with poly-L-lysine (Sigma) were infected with YFV-17D or DENV-NGC at an MOI of 0.1 or 1, respectively. At 24 hpi, cells were collected in 0.05% trypsin (Gibco) and 50% of the cell suspension of was transferred with fresh medium to a new well coated with poly-L-lysine. ROS production was measured at 48 hpi with the Mitochondrial Superoxide Detection Kit (Abcam, ab219943). Cells were loaded for 30 min with MitoROS 580 and 0.1 ug/ml Hoechst 33342 (Thermo) in growth medium without phenol red according to manufacturer instructions and imaged (20X, 10x10 tiled images) live using a Nikon Eclipse Ti2 inverted epifluorescence microscope fitted with a Hamamatsu ORCA-FusionBT and a Lumencor Spectra X excitation source. Cell monolayers were fixed with 100% ice-cold methanol, blocked for 10 min in PBS with 5%FBS, stained with dsRNA (J2) mouse antibody (#76651, Cell Signaling) and goat anti-Mouse IgG Alexa Fluor 647 secondary antibody (Thermo) in PBS 0.5% Triton X-100 5% FBS, and counter stained with HCS CellMask Blue Stain in PBS. Fixed cells were imaged and aligned with the live images using Nikon NIS-Elements Advanced Research software image registration of the Hoechst channel. CellProfiler was used to quantitate MitoROS 580 and J2 signal intensities of cells (N > 2500) in each experimental replicate (N = 3), as previously described [74].

### Western blot

We washed and lysed cells in SDS extraction buffer (50 mM Tris pH 7.4, 150 mM sodium chloride, 1mM EDTA, 2% SDS) supplemented with complete protease inhibitor (Sigma Aldrich). Lysates were agitated at 95˚C for 10 minutes. We quantified protein concentration using the DC Protein assay (Bio-Rad) per the manufacturer’s protocol before diluting lysates 1:4 in sample buffer (90% 4X Protein Loading Buffer (LiCor), 10% DTT (Sigma Aldrich)). We heated diluted samples at 70˚C for 10 minutes. We resolved 12 µg of cell lysate on 4–12% Bis-Tris gels (Thermo Fisher Scientific) and transferred to Nitrocellulose or PVDF membranes (Invitrogen) using the iBlot 2 Transfer System (Invitrogen). We blocked the membrane for 1 hour with Intercept Blocking Buffer (LiCor) followed by overnight incubation at 4˚C in primary antibody diluted in blocking buffer. We washed membranes three times for 5 minutes with PBST before incubating at room temperature for 1 hour in secondary antibody diluted in blocking buffer. We washed membranes several times with PBST before imaging on the LiCor Odyssey CLx Imager.

### siRNA treatment

We used the following siRNAs: Horizon ON-TARGETplus SMARTpool siRNAs specific against DRP1 (L-012092), MAVS (L-024237), MFN2 (L-012961), and STING (L-024333). We transfected HepG2 cells with 20 mmol siRNA using the Lipofectamine RNAiMAX reagent (Life Technologies) according to the manufacturer’s protocol. We incubated cells with siRNA for 48 hours prior to infection and downstream analysis.

### Metabolite sample preparation

We washed HepG2 cells with a 0.9% sodium chloride solution at room temperature. To quench cellular metabolism, we added ice-cold LCMS-grade methanol (Fisher Chemical) and incubated on ice for 5 minutes. We added an equal volume of ice-cold LCMS-grade water (Fisher Chemical) before scraping cells and transferring samples to polypropylene tubes which were stored at -80˚C until analysis. We randomized samples across plates to account for minor variations in sample collection time. The aqueous fraction was taken for LCMS analysis and diluted as needed in a 1:1 mixture of methanol and water to balance signal intensity.

### Liquid chromatography mass spectrometry

We purchased tributylamine and all synthetic molecular references from Millipore Sigma. We purchased LCMS grade water, methanol, isopropanol and acetic acid through Fisher Scientific.

We analyzed aqueous metabolites using a combination of two analytical methods with opposing ionization polarities [75,76]. Both methodologies utilized a LD40 XR UHPLC (Shimadzu Co.) system for separation. Negative mode samples were separated on a Waters Atlantis T3 column (100Å, 3 µm, 3 mm X 100 mm) and eluted using a binary gradient from 5 mM tributylamine, 5 mM acetic acid in 2% isopropanol, 5% methanol, 93% water (v/v) to 100% isopropanol over 5 minutes. Signals were detected using a 5500 QTrap mass spectrometer (AB Sciex Pte. Ltd.). Two distinct MRM pairs in negative mode were used for each metabolite. Positive mode method samples were separated across a Phenomenex Kinetex F5 column (100 Å, 2.6 µm, 100 x 2.1 mm) and eluted with a gradient from 0.1% formic acid in water to 0.1% formic acid in acetonitrile over 5 minutes. Signals were detected using a 6500 + QTrap mass spectrometer (AB Sciex Pte. Ltd.).

All signals were integrated using SciexOS 3.1 (AB Sciex Pte. Ltd.). Signals with greater than 50% missing values for a specific tissue set were discarded and remaining missing values were replaced with the lowest registered signal value. Where appropriate, signals with a QC coefficient of variance greater than 30% were discarded. Metabolites with multiple MRMs were quantified with the higher signal to noise MRM. The filtered dataset of the negative mode aqueous metabolites was total sum normalized after initial filtering. The positive mode aqueous metabolomics dataset was scaled and combined with the negative mode aqueous metabolite dataset using a common signal for serine. A Benjamini-Hochberg method for correction for multiple comparisons was imposed where indicated.

### Seahorse extracellular flux analysis

We seeded Hep2G cells at 1 × 10^4^ cells per well in a XFe96 tissue culture plate (Agilent Technologies) and incubated for 24 hours in cDMEM. We infected cells with mock, YFV-17D (MOI 0.1) or DENV2 (MOI 1) as previously described. At 12-, 24-, and 48-hours post infection we performed extracellular flux analysis to interrogate the impacts of infection on mitochondrial function and glycolysis Prior to extracellular flux analyses, we washed cells twice with 200 μL of assay medium (minimal DMEM with 25 mM glucose, 2 mM sodium pyruvate, and 2 mM L-glutamine (Agilent Technologies)). We incubated cells in 180 µ L Assay medium for 1 hour at 37˚C in a non-CO_2_ incubator. We measured oxygen consumption rate (OCR) rate at baseline and following injection of oligomycin A1 (2 μM, MilliporeSigma), fluoro-carbonyl cyanide phenylhydrazone (FCCP; 2 μM; Cayman Chemical), and rotenone/antimycin (0.5 μM final concentration for both; MilliporeSigma). We used corresponding extracellular acidification rates (ECAR) as surrogate markers of changes in glycolytic flux. We performed all extracellular flux assays on the Seahorse XFe96 Analyzer (Agilent Technologies).

### Flow cytometry

We used the following cellular dyes: TMRE (Abcam), MitoSOX (Abcam), Peroxynitrite Green (Abcam), and Annexin V/PI (Thermo Fisher Scientific) according to the respective manufacturer’s protocol. Following staining, we washed cells several times with PBS and harvested as a single cell solution using trypsin. When appropriate, we also stained cells with the Live/Dead Fixable Aqua Dead Cell Stain (Thermo Fisher Scientific) for 20 minutes on ice to identify the live cell population. Flow cytometry analysis was performed on a BD LSR Fortessa X-20 flow cytometer.

### ATP quantitation

We extracted ATP from cells using the boiling water method to minimize ATPase depletion. We washed HepG2 cells twice with PBS and then added boiling highly purified water (ThermoFisher), triturating to lyse cells and boiling for several more minutes to completely inactivate ATPases. We stored samples at -80˚C until analysis. We measured in ATP in each sample in triplicate using an ATP Determination Kit (Thermo Fisher Scientific) according to the manufacturer’s protocol, analyzing all samples in one assay to maximize reproducibility.

### Small molecule inhibitors

We used MnTBAP (Sigma Aldrich), L-NAME or N-acetylcysteine (Selleck Chemicals). We prepared a 50 mM stock solution of MnTBAP in DMSO (ATCC). Immediately before use, we prepared a reduced working solution by diluting the MnTBAP to a final concentration of 1mM in PBS/4% NaOH [2.5M] (Sigma Aldrich). We added 100 µ M of the reduced working solution to cells in a drop-wise fashion to minimize cell death. For this treatment, the vehicle control consisted of an equal volume of DMSO in PBS/4% NaOH [2.5M]. Alternately, we treated cells with 5mM L-NAME or 10mM N-acetylcysteine (NAC) in sterile water and an equal volume of water was used as a vehicle control for these experiments. For all inhibitors, we retreated cells every 24 hours to maintain high inhibitor concentration.

### qRT-PCR

HepG2 cells were seeded in 24 well plates and infected with YFV-17D or Asibi (MOI = 0.01 and 0.1). At the times specified, total RNA was extracted with RNeasy Plus Mini Kit (Qiagen) per manufacturer’s recommendations. 500ng of purified RNA was converted into cDNA using the SuperScript VILO cDNA Master Mix (Thermo Fisher Scientific) prior to qRT-PCR with the Luna Universal Probe qPCR Master Mix (NEB) following manufacturer’s recommendations. Standards were generated by 1/10 serial dilutions of the YFV-17D molecular clone, where a standard curve was generated to calculate genome copy numbers for each sample.

### SDD-AGE for MAVS oligomerization

Methods were performed as previously described [43,77] with the following modifications. HepG2 cells were seeded in 6 well plates and infected with YFV-17D (MOI = 0.1) for 1hr. Complete media containing 100 µ M of MnTBAP or DMSO vehicle control was added 1 hpi and lysates harvested 48 hpi with 100 µ L of NP40 lysis buffer (Boston BioProducts) with cOmplete EDTA-free Protease Inhibitor Cocktail (Roche) per well and incubated on ice for 20 min. Following cellular debris removal by centrifugation at 21,000 x g, 50 µ L of clarified cell lysate was treated with 100 µ M ß-ME for 30 min. 2X SDD-AGE sample buffer (2% SDS, 10% glycerol, 0.02% bromophenol blue in 0.5X TBE) was added into 50 µ L of each ß -ME treated and untreated sample, loaded onto a 1.5% agarose gel prepared with 1X TBE buffer and resolved by vertical electrophoresis (70V, 120mA, 1 hour) in SDD-AGE running buffer (1X TBE and 0.1% SDS). In addition, the remaining sample post loading in the 1.5% agarose gel was boiled at 95^o^C and resolved by SDS-PAGE. The SDD-AGE gels were transferred (1.3A, 25V for 20 min) and blocked with 5% milk in TBST for 1 h. Primary antibodies to detect MAVS (Santa Cruz Biotechnology), YFV NS3 (GeneTex) and Vinculin (Sigma) were incubated overnight and appropriate goat anti-mouse and -rabbit HRP conjugated secondary antibodies (Sigma) added for 1 h prior to detection by chemiluminescence with SuperSignal West Femto (Thermo Fisher Scientific).

### Full-length molecular clones

All work was approved by the RML Institutional Biosafety Committee and separately by the NIH Dual Use Research Committee. Full length YFV Asibi virulent strain (MT093734.1) and 17D vaccine strain (MT114401.1) infectious clones have been previously described [78,79] and are designated pACNR-2015FLYF-Asibi and pACNR-2015FLYF-17Da respectively. The E.coli strain MC1061 was used to amplify these clones. After amplification and purification, plasmid DNA was linearized using AflII, followed by clean up and in vitro transcription using the mMESSAGE mMACHINE SP6 transcription kit. The virus stock was produced in Huh7.5 cells using 10μg of RNA essentially as described [80]. Cells of the electroporation reaction were seeded in a T-175 flask. At 4–6 hours post-electroporation, the media was changed and at 20–24 hours post-electroporation, media was replaced with serum free DMEM. Virus stocks were obtained by harvesting the media after an additional 6–8 hours and aliquots were stored at -80^o^C.

### Human monocyte-derived dendritic cells

Human monocyte-derived dendritic cells were prepared as previously described [81]. Briefly, we isolated peripheral blood mononuclear cells from human buffy coats (BioIVT) by centrifugation through a Ficoll-Paque Plus density gradient (Cytiva). Cells were enriched for CD14^+^ monocytes using a RossetteSep Monocyte Enrichment Kit (StemCell Technologies). Monocytes were resuspended at 1x10^6^ cells/mL in DC medium [RPMI + Glutamax (Invitrogen), 5% FBS, 15 mM Hepes, 0.1 mM nonessential amino acids, 1 mM sodium pyruvate, 100 units/mL penicillin and 100 μg/mL streptomycin] containing IL-4 (20 ng/mL) and GM-CSF (20 ng/ml) (PeproTech) and cultured for 5d with replacement of half of the medium and addition of fresh cytokines every other day. Nonadherent DC were harvested by centrifugation and suspended in DC medium. The resulting cells were determined to be > 95% CD11c^+^/CD209^+^ by flow cytometry.

### Bulk RNA-seq of human DCs

We plated human monocyte-derived DCs at 2x10^6^ cells/well and mock-infected or infected with YFV-17D at an MOI of 0.1 in a small volume for 1 hour at 37˚C, 5% CO_2_ with occasional gentle rocking. We added fresh DC medium to the wells to the desired volume and MnTBAP (50 µ M) or vehicle control was added to the appropriate wells at 0 and 24 h post-infection. At 48 hpi, we harvested hDCs by centrifugation, and lysed the cells with 1 mL of TRIzol reagent/sample (Thermo Fisher Scientific), and samples were stored at -80˚C before extraction and bulk RNA-seq. We collected cell supernatants at 0 and 48 hpi for cytokine and viral plaque assays.

For RNA extraction, we mixed the samples with 200 µ L of 1-bromo-3-chloropropane (Sigma Aldrich) and centrifuged at 4˚C for 15 minutes at 16,000 x g. We removed the aqueous phase and passed it through a QIAshredder column (Qiagen) at 21,000 x g for 2 minutes. RNA was extracted using the Qiagen AllPrep DNA/RNA system. An additional on-column DNase I treatment was performed for all extractions. We assessed RNA quality using the Agilent 2100 Bioanalyzer RNA 6000 Pico kit (Agilent Technologies) and quantified the RNA using the Quant-it RiboGreen RNA assay (Thermo Fisher Scientific).

Following RNA extraction, two hundred nanograms of RNA was used as the input for the Illumina stranded mRNA prep, ligation kit (Illumina) to generate sequencing libraries following the manufacturer’s protocol. The final libraries were analyzed using the Agilent bioanalyzer and the libraries were quantified using the Kapa SYBR FAST Universal qPCR kit for Illumina sequencing (Kapa Biosystems). The individual libraries were diluted to 6 nM and 2 uL of each added to a library pool. The library pool was denatured and diluted to a 10 pM stock and paired-end 2 x 75 cycle sequencing carried out on the MiSeq using a Nano V2 flow cell and 300 cycle chemistry. Following the sequencing run, reads per microliter mapping to human genes were determined for each library. The library pool was rebalanced and quantified using the Kapa SYBR FAST Universal qPCR kit for Illumina sequencing. The library pool was diluted to 9 pM and paired-end 2 x 75 cycle sequencing carried out on the MiSeq using a Nano V2 flow cell and 300 cycle chemistry. The final library pool containing 6 nM of each library was sent to the National Cancer Institute, Center for Cancer Research Sequencing Facility (NCI CCR-SF) for further sequencing. The samples were paired-end 2 x 100 cycle sequenced using a NovaSeq 6000 instrument and SP flow cell and 300 cycle chemistry.

Following sequencing, Raw fastq files were trimmed to remove adapters and low-quality bases using Cutadapt v1.18 before alignment to the GRCh38 reference genome and the Gencode v42 genome annotation using STAR v2.7.9a [82]. PCR duplicates were marked using the MarkDuplicates tool from the Picard v3.1.0 (https://broadinstitute.github.io/picard/) software suite. Raw gene counts were generated using RSEM v1.3.3 [83] and were filtered to include genes with >=1 count per million (CPM) in at least 2 samples. Raw datasets have been deposited with Gene Expression Omnibus (GEO) accession number GSE293880.

Differential expression was evaluated using limma with TMM normalization [84]. For all differential expression comparisons, pre-ranked geneset enrichment analysis (GSEA) [85] was performed using the Reactome [86] and MitoCarta v3.0 [87].

### Statistical analysis

All graphical values are shown as mean ± standard deviation. We performed statistical analyses using GraphPad Prism 10. Ordinary one-way or two-way ANOVAs with Dunnett’s, Sidak’s or Tukey’s multiple comparisons tests were performed, when appropriate, to test statistical significance between means. P-values less that 0.05 were considered statistically significant.

## Supporting information

S1 Fig Differential mitochondrial morphodynamics are consistent across multiple cell types. **(A)** Growth curve quantifying the viral titer and ELISA quantification of IFNβ secreted by Huh7 cells infected with YFV-17D (MOI 0.1) or DENV2 (MOI 0.1) over a 72 h period. **(B)** IF of mitochondrial morphology in Huh7 cells following infection with mock, DENV2 (MOI 1), YFV-17D (MOI 0.1), or YFV-Asibi (MOI 1) for 48 h. Nuclei are stained with DAPI in blue, mitochondria are stained with TOM20 in green, and infected cells are stained with NS3 in red.
(TIFF)

S2 Fig siRNA treatment results in knockdown of host proteins. Western blot demonstrating reduced protein expression following siRNA treatment of HepG2 cells with a control nontargeting (siNT) siRNA or specific for **(A)** siMAVS, **(B)** siSTING, **(C)** siMFN2, or **(D)** siDRP1. Treatment with siRNA resulted in significant knockdown of the respective protein.
(TIFF)

S3 Fig Very few metabolites are differentially abundant between YFV-17D and mock infected cells at 24 and 36 hpi. (**A and B**) Volcano plots showing significantly differentially abundant metabolites between YFV-17D infected Hep G2 cells and mock-infected HepG2 cells at **A** 24 hpi and **B** 36 hpi.
(TIFF)

S4 Fig YFV-17D upregulates glycolysis between 24 and 48 hpi. **(A)** Metabolomic heatmap comparing the abundance of metabolites from the glycolytic pathway, pentose-5-phosphate pathway, and nucleotides following infection with mock, YFV-17D (MOI 0,1), YFV-Asibi (MOI 1), or DENV2 (MOI 1) in HepG2 cells. **(B)** Schematic of the glycolysis pathway with metabolites significantly downregulated in YFV-17D infection at 48 hpi shown in blue and metabolites significantly upregulated in YFV-17D infection at 48 hpi shown in red. **(C)** Schematic of the glycolysis and pentose-5-phosphate pathway with metabolites significantly downregulated in YFV-17D infection at 48 hpi shown in in blue and metabolites significantly upregulated in YFV-17D infection at 48 hpi shown in red. **(D)** Quantification of the media acidification rate obtained during the Seahorse XF mito stress test in mock, YFV-17D, or DENV2 infected HepG2 cells over a 48 h period. Values represent the mean ± SD (n = 12). Statistical significance was assessed using two-way ANOVA followed by Dunnett’s multiple comparisons test **(D)**. * p < 0.05, *** p < 0.001, **** p < 0.0001.
(TIFF)

S5 Fig Loss of mitochondrial membrane potential and IFNβ expression is related to rapid dynamics of YFV-17D replication. **(A)** Growth curve quantifying viral titer and ELISA quantification of IFNβ secreted by HepG2 cells infected with YFV-17D or YFV-Asibi at 2 MOIs. **(B)** Quantification of mitochondrial membrane potential and **(C)** genome copies from the experiment in **(A).** Values represent the mean ± SD (A, B: n = 5; C: n = 3). Statistical significance was assessed using two-way ANOVA followed by Sidak **(A)** or Dunnett’s **(B)** post hoc test for multiple groups, or ordinary one-way ANOVA followed by Sidak’s post hoc test for multiple groups **(C)**. * p < 0.05, ** p < 0.01, *** p < 0.001, **** p < 0.0001.
(TIFF)

S6 Fig YFV-17D does not induce apoptosis until late timepoints. **(A)** Representative flow cytometry dot plots quantifying Annexin V and propidium iodide in mock, YFV-17D (MOI 0.1), and DENV2 (MOI 1) infected HepG2 cells at 24hpi, 48hpi, and 72hpi. **(B)** Quantification of the percentage of apoptotic cells in mock, YFV-17D, and DENV2 infected cells at 24hpi, 48hpi, and 72hpi. Values represent the mean ± SD (n = 3). Statistical significance was assessed using two-way ANOVA followed by Dunnett’s multiple comparisons test **(D)**. **** p < 0.0001.
(TIFF)

S7 Fig Peroxynitrite appears to be the main source of ROS in YFV-17D infection. **(A)** ELISA quantification of IFNβ secreted by HepG2 cells infected with YFV-17D and treated with NAC [10nM] at 0hpi or 24hpi. **(B)** Flow cytometric quantification of peroxynitrite staining in mock, YFV-17D, and DENV2 infected HepG2 cells treated with MnTBAP or a vehicle control for 48 hours. **(C)** ELISA quantification of IFNβ secreted by HepG2 cells infected with YFV-17D and treated with L-NAME [5mM] at 0hpi with corresponding growth curve quantifying the viral titer. **(D)** Schematic showing the proposed mechanism by which both MnTBAP and L-NAME inhibit peroxynititre formation thereby blocking type I IFN production. Values represent the mean ± SD (n = 3). Statistical significance was assessed using two-way ANOVA followed by Dunnett’s multiple comparisons test **(A)**, ordinary one-way ANOVA followed by Tukey’s multiple comparisons test **(B)**, or two-way ANOVA followed by Sidak’s multiple comparisons test **(C)**. * p < 0.05, ** p < 0.01, **** p < 0.0001.
(TIFF)

S8 Fig Loss of mitochondrial membrane potential is independent of type-I IFN signaling. Quantification of mitochondrial membrane potential (TMRE) in HepG2 cells infected with mock or YFV-17D (MOI 0.1) treated with **(A)** MnTBAP or **(B)** siMAVS to inhibit type-I IFN signaling. Values represent the mean ± SD (n = 3). Statistical significance was assessed using two-way ANOVA followed by Tukey’s post hoc test for multiple groups. ** p < 0.01, *** p < 0.001, **** p < 0.0001.
(TIFF)

S9 Fig MnTBAP treatment ameliorates effects of YFV-17D infection. **(A)** Heatmap comparing the expression of oxidative stress genes in mock and YFV-17D (MOI 0.1) infected human DCs treated with and without MnTBAP.
(TIFF)

S1 Table Sequence differences between YFV-17D lab stock and YFV-17D vaccine strain (MT114401.1) molecular clone. (DOCX)

S2 Table Antiviral immune pathway analysis highlighting DEGs in human monocyte-derived DCs at 48 hpi with YFV-17D and treated with MnTBAP. (DOCX)
