# Supplementary figures and images for: Mitochondrial hyperactivity and reactive oxygen species drive innate immunity to the yellow fever virus-17D live-attenuated vaccine
Source: PLoS Pathog. 2025 Apr 21;21(4):e1012561. doi: 10.1371/journal.ppat.1012561 (PMC12052391; doi:10.1371/journal.ppat.1012561)

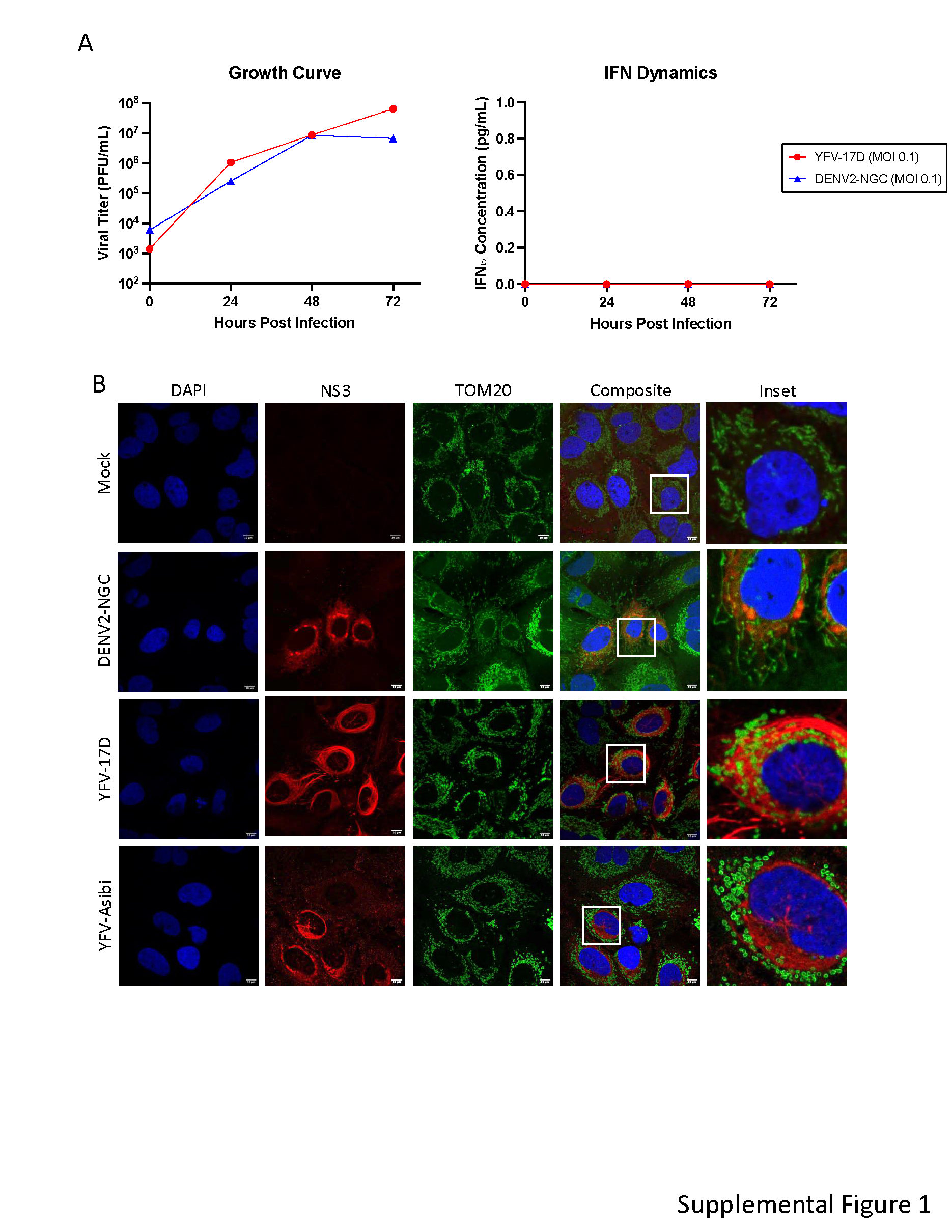

Supplement: S1 Fig — (A) Growth curve quantifying the viral titer and ELISA quantification of IFNβ secreted by Huh7 cells infected with YFV-17D (MOI 0.1) or DENV2 (MOI 0.1) over a 72 h period. (B) IF of mitochondrial morphology in Huh7 cells following infection with mock, DENV2 (MOI 1), YFV-17D (MOI 0.1), or YFV-Asibi (MOI 1) for 48 h. Nuclei are stained with DAPI in blue, mitochondria are stained with TOM20 in green, and infected cells are stained with NS3 in red. (TIFF) [file ppat.1012561.s001.tiff]

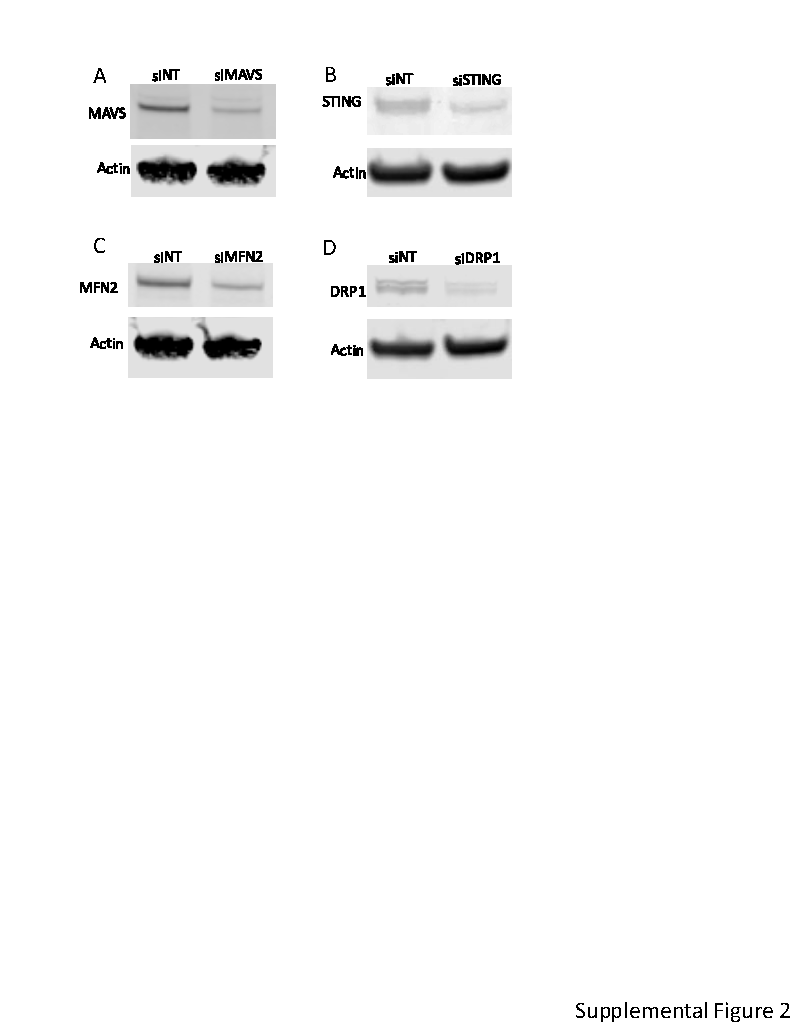

Supplement: S2 Fig — Western blot demonstrating reduced protein expression following siRNA treatment of HepG2 cells with a control nontargeting (siNT) siRNA or specific for (A) siMAVS, (B) siSTING, (C) siMFN2, or (D) siDRP1. Treatment with siRNA resulted in significant knockdown of the respective protein. (TIFF) [file ppat.1012561.s002.tiff]

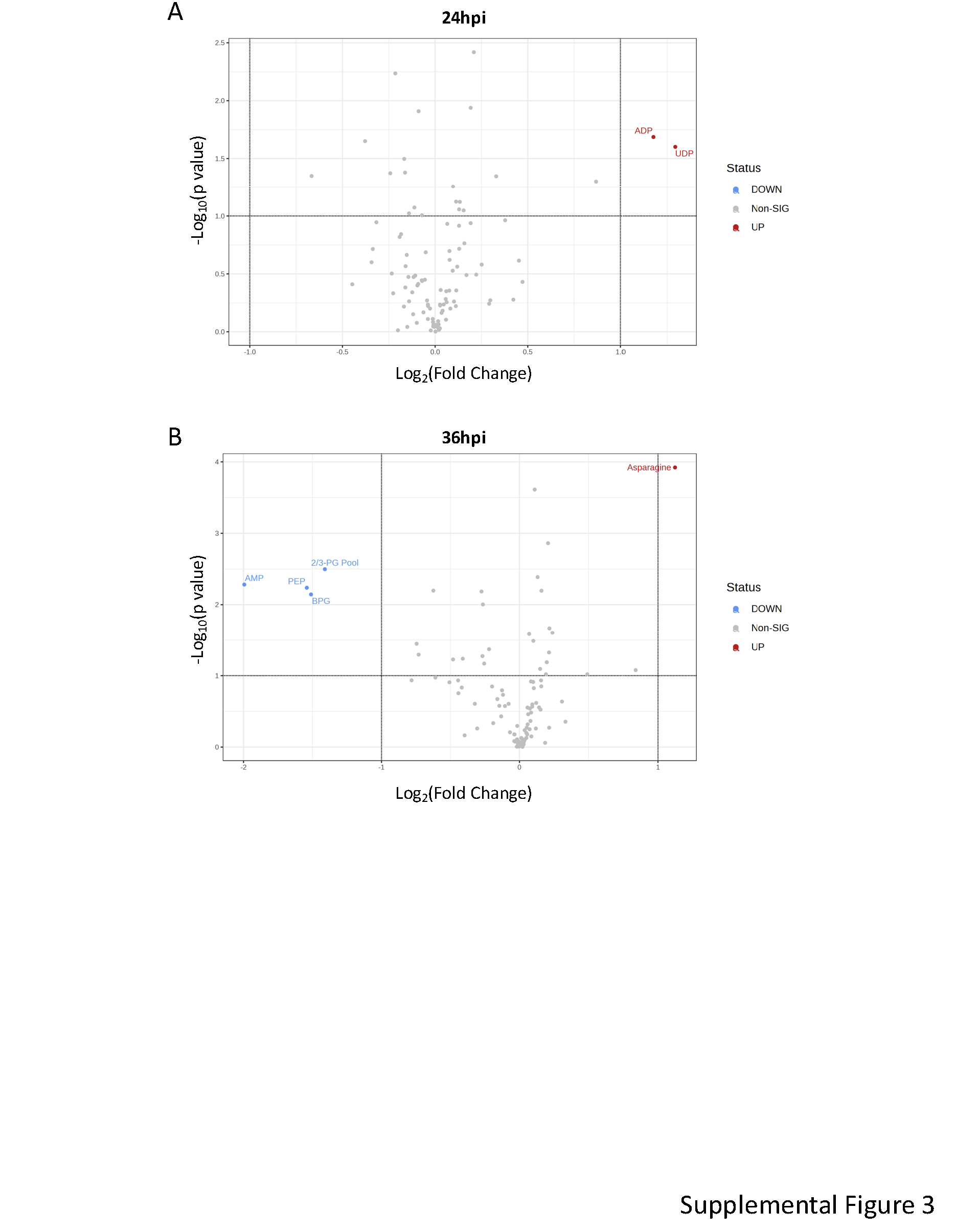

Supplement: S3 Fig — (A and B) Volcano plots showing significantly differentially abundant metabolites between YFV-17D infected Hep G2 cells and mock-infected HepG2 cells at A 24 hpi and B 36 hpi. (TIFF) [file ppat.1012561.s003.tiff]

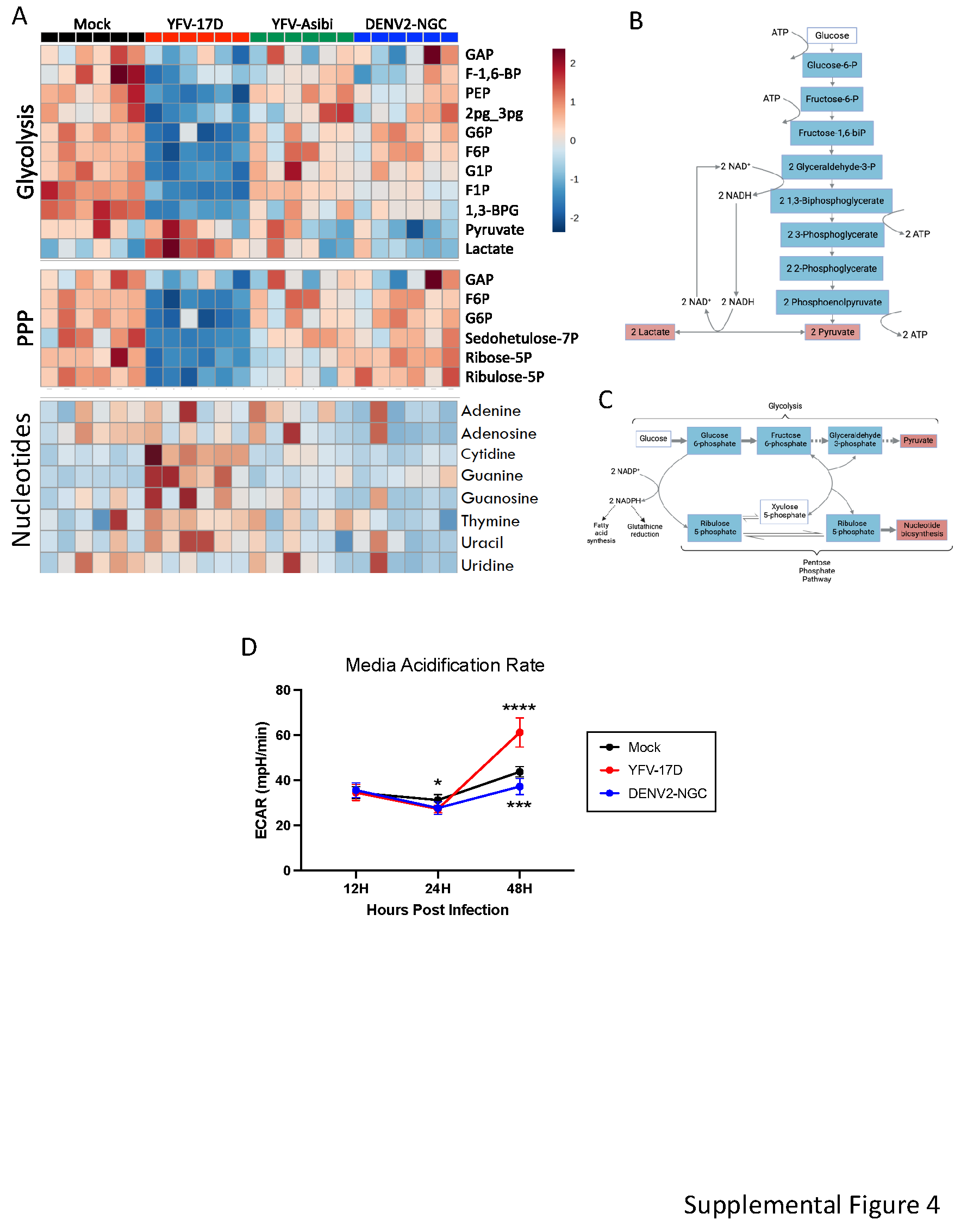

Supplement: S4 Fig — (A) Metabolomic heatmap comparing the abundance of metabolites from the glycolytic pathway, pentose-5-phosphate pathway, and nucleotides following infection with mock, YFV-17D (MOI 0,1), YFV-Asibi (MOI 1), or DENV2 (MOI 1) in HepG2 cells. (B) Schematic of the glycolysis pathway with metabolites significantly downregulated in YFV-17D infection at 48 hpi shown in blue and metabolites significantly upregulated in YFV-17D infection at 48 hpi shown in red. (C) Schematic of the glycolysis and pentose-5-phosphate pathway with metabolites significantly downregulated in YFV-17D infection at 48 hpi shown in in blue and metabolites significantly upregulated in YFV-17D infection at 48 hpi shown in red. (D) Quantification of the media acidification rate obtained during the Seahorse XF mito stress test in mock, YFV-17D, or DENV2 infected HepG2 cells over a 48 h period. Values represent the mean ± SD (n = 12). Statistical significance was assessed using two-way ANOVA followed by Dunnett’s multiple comparisons test (D). * p < 0.05, *** p < 0.001, **** p < 0.0001. (TIFF) [file ppat.1012561.s004.tiff]

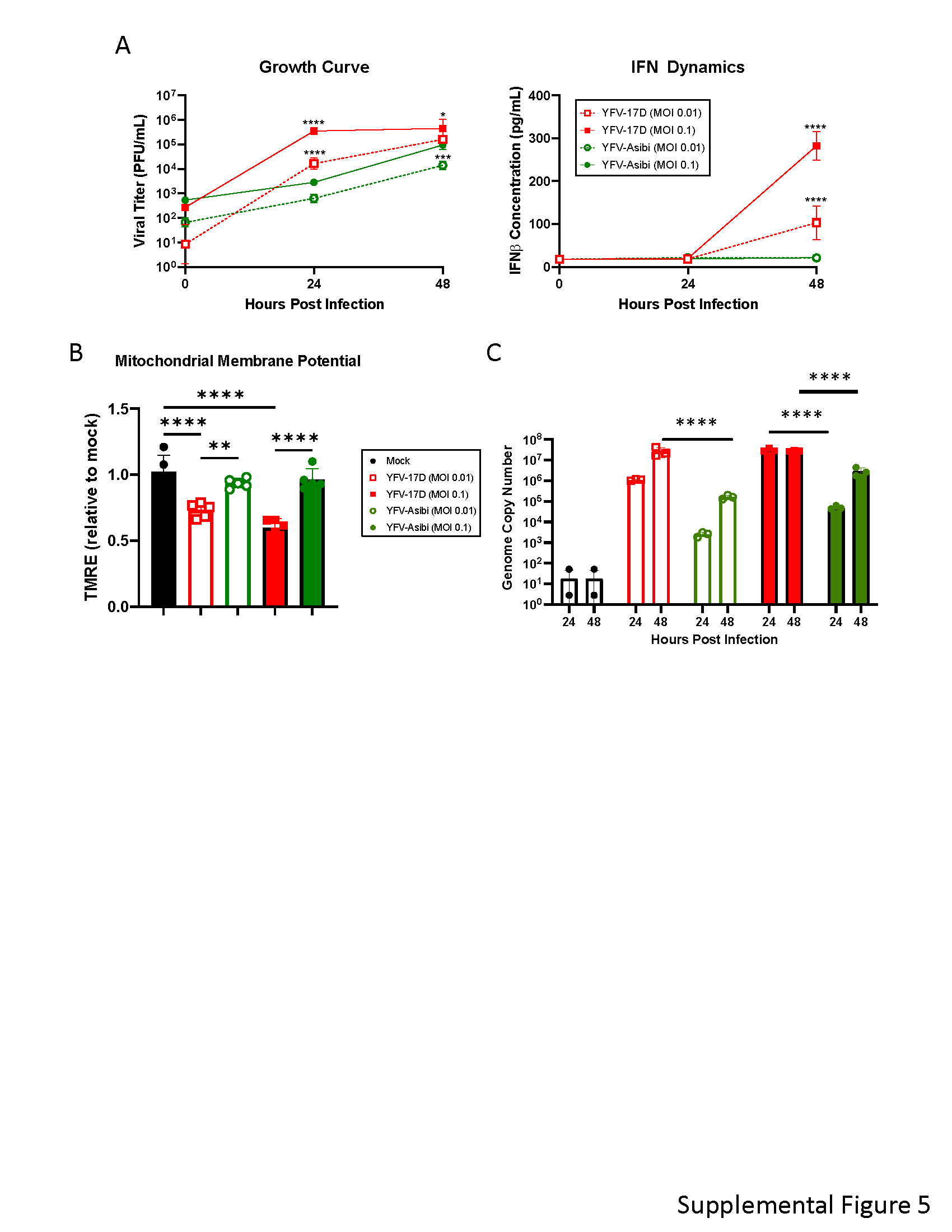

Supplement: S5 Fig — (A) Growth curve quantifying viral titer and ELISA quantification of IFNβ secreted by HepG2 cells infected with YFV-17D or YFV-Asibi at 2 MOIs. (B) Quantification of mitochondrial membrane potential and (C) genome copies from the experiment in (A). Values represent the mean ± SD (A, B: n = 5; C: n = 3). Statistical significance was assessed using two-way ANOVA followed by Sidak (A) or Dunnett’s (B) post hoc test for multiple groups, or ordinary one-way ANOVA followed by Sidak’s post hoc test for multiple groups (C). * p < 0.05, ** p < 0.01, *** p < 0.001, **** p < 0.0001. (TIFF) [file ppat.1012561.s005.tiff]

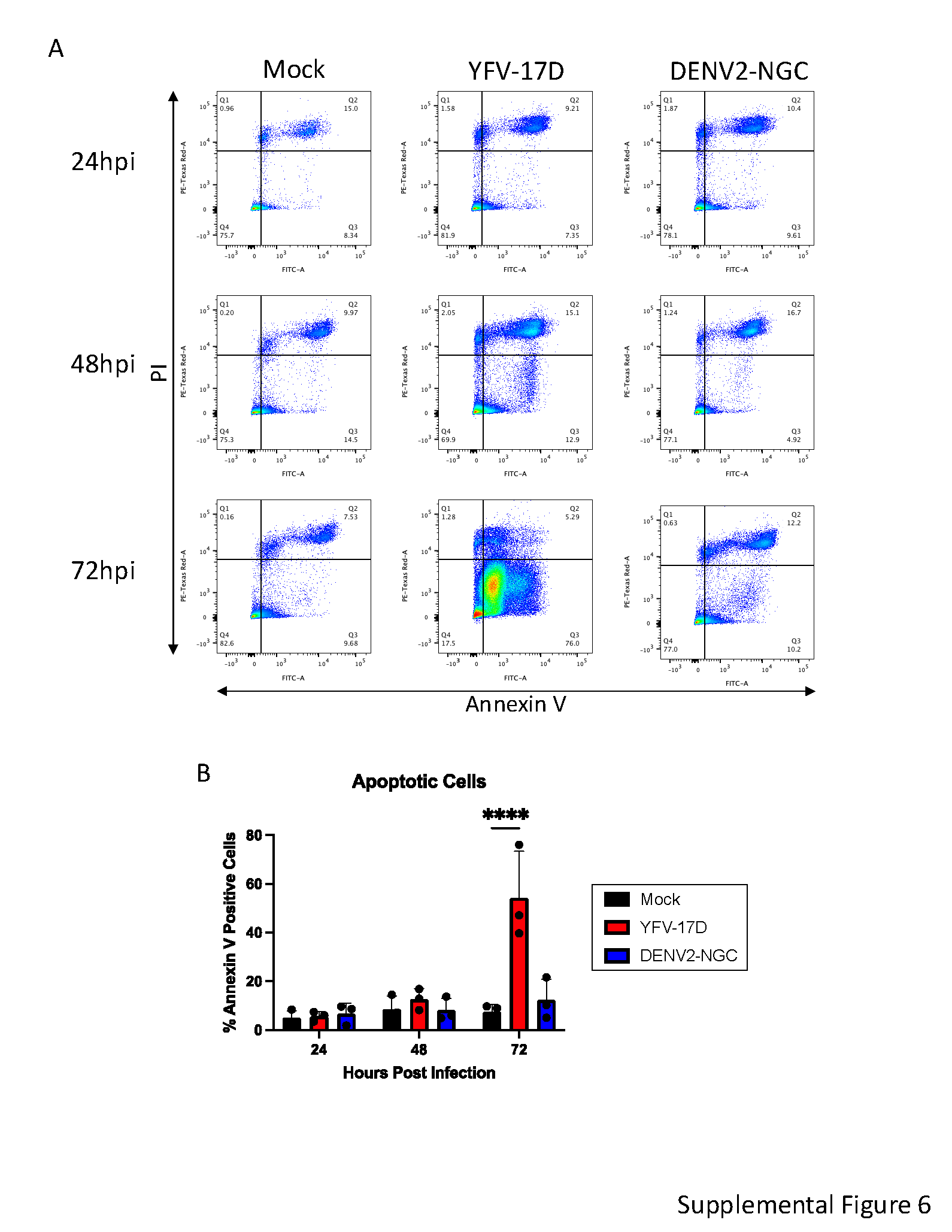

Supplement: S6 Fig — (A) Representative flow cytometry dot plots quantifying Annexin V and propidium iodide in mock, YFV-17D (MOI 0.1), and DENV2 (MOI 1) infected HepG2 cells at 24hpi, 48hpi, and 72hpi. (B) Quantification of the percentage of apoptotic cells in mock, YFV-17D, and DENV2 infected cells at 24hpi, 48hpi, and 72hpi. Values represent the mean ± SD (n = 3). Statistical significance was assessed using two-way ANOVA followed by Dunnett’s multiple comparisons test (D). **** p < 0.0001. (TIFF) [file ppat.1012561.s006.tiff]

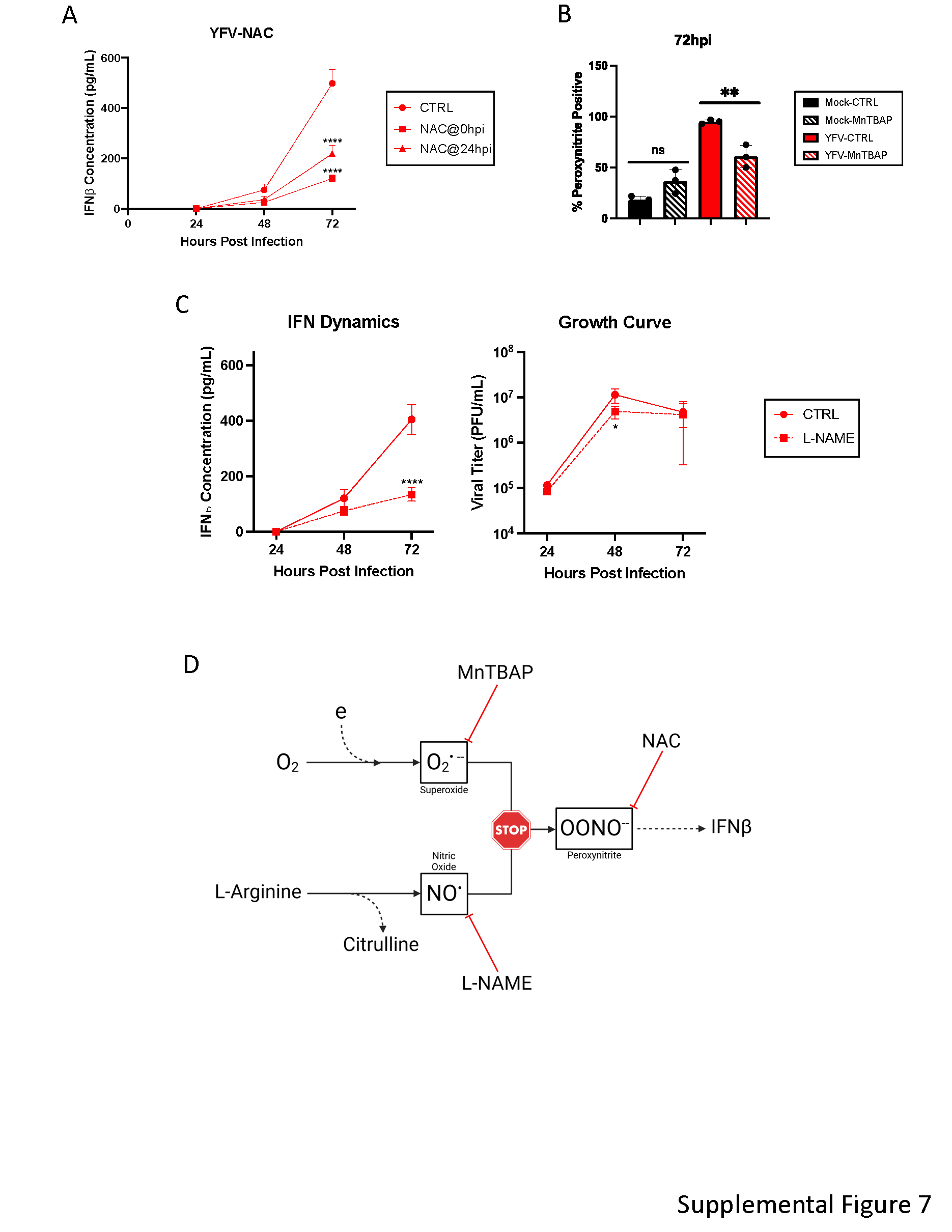

Supplement: S7 Fig — (A) ELISA quantification of IFNβ secreted by HepG2 cells infected with YFV-17D and treated with NAC [10nM] at 0hpi or 24hpi. (B) Flow cytometric quantification of peroxynitrite staining in mock, YFV-17D, and DENV2 infected HepG2 cells treated with MnTBAP or a vehicle control for 48 hours. (C) ELISA quantification of IFNβ secreted by HepG2 cells infected with YFV-17D and treated with L-NAME [5mM] at 0hpi with corresponding growth curve quantifying the viral titer. (D) Schematic showing the proposed mechanism by which both MnTBAP and L-NAME inhibit peroxynititre formation thereby blocking type I IFN production. Values represent the mean ± SD (n = 3). Statistical significance was assessed using two-way ANOVA followed by Dunnett’s multiple comparisons test (A), ordinary one-way ANOVA followed by Tukey’s multiple comparisons test (B), or two-way ANOVA followed by Sidak’s multiple comparisons test (C). * p < 0.05, ** p < 0.01, **** p < 0.0001. (TIFF) [file ppat.1012561.s007.tiff]

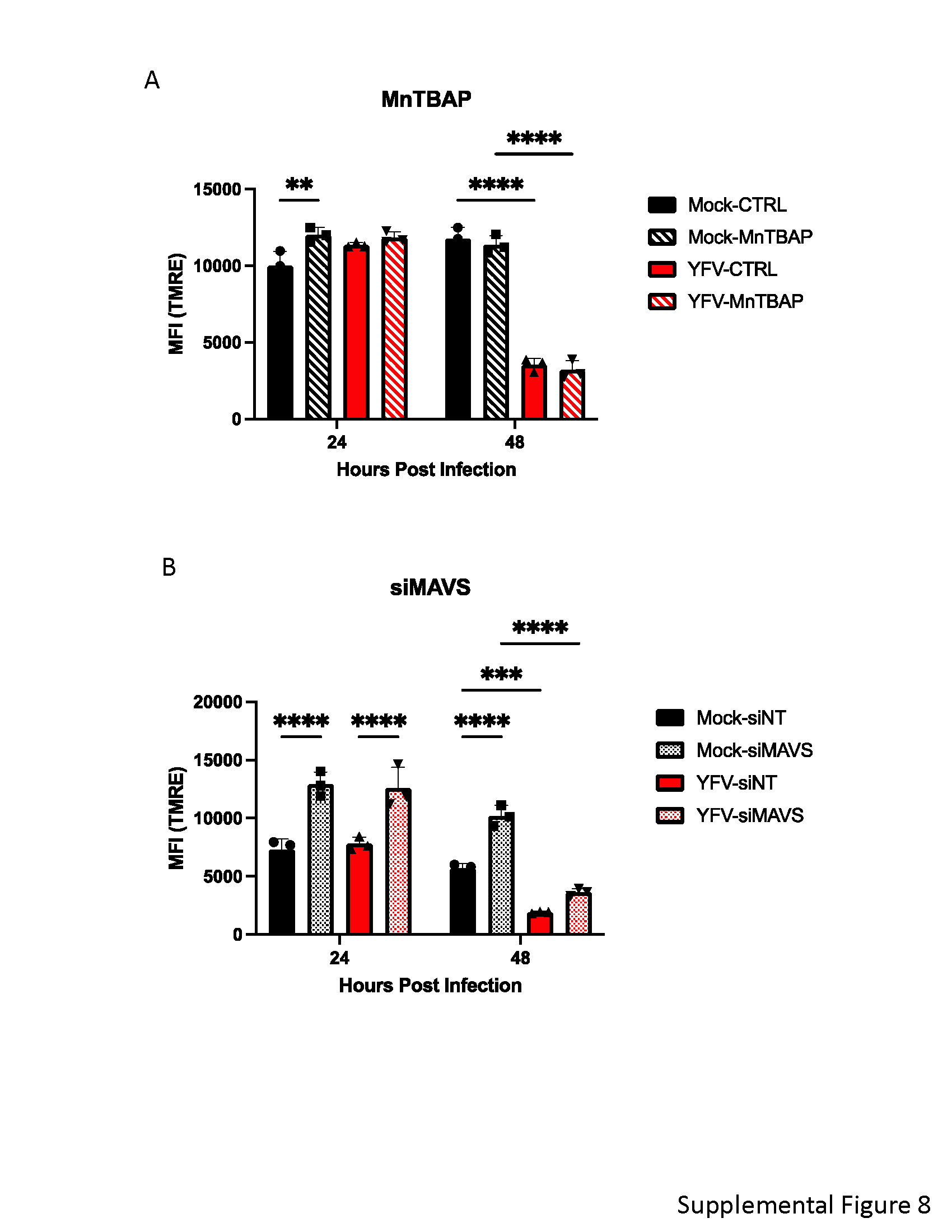

Supplement: S8 Fig — Quantification of mitochondrial membrane potential (TMRE) in HepG2 cells infected with mock or YFV-17D (MOI 0.1) treated with (A) MnTBAP or (B) siMAVS to inhibit type-I IFN signaling. Values represent the mean ± SD (n = 3). Statistical significance was assessed using two-way ANOVA followed by Tukey’s post hoc test for multiple groups. ** p < 0.01, *** p < 0.001, **** p < 0.0001. (TIFF) [file ppat.1012561.s008.tiff]

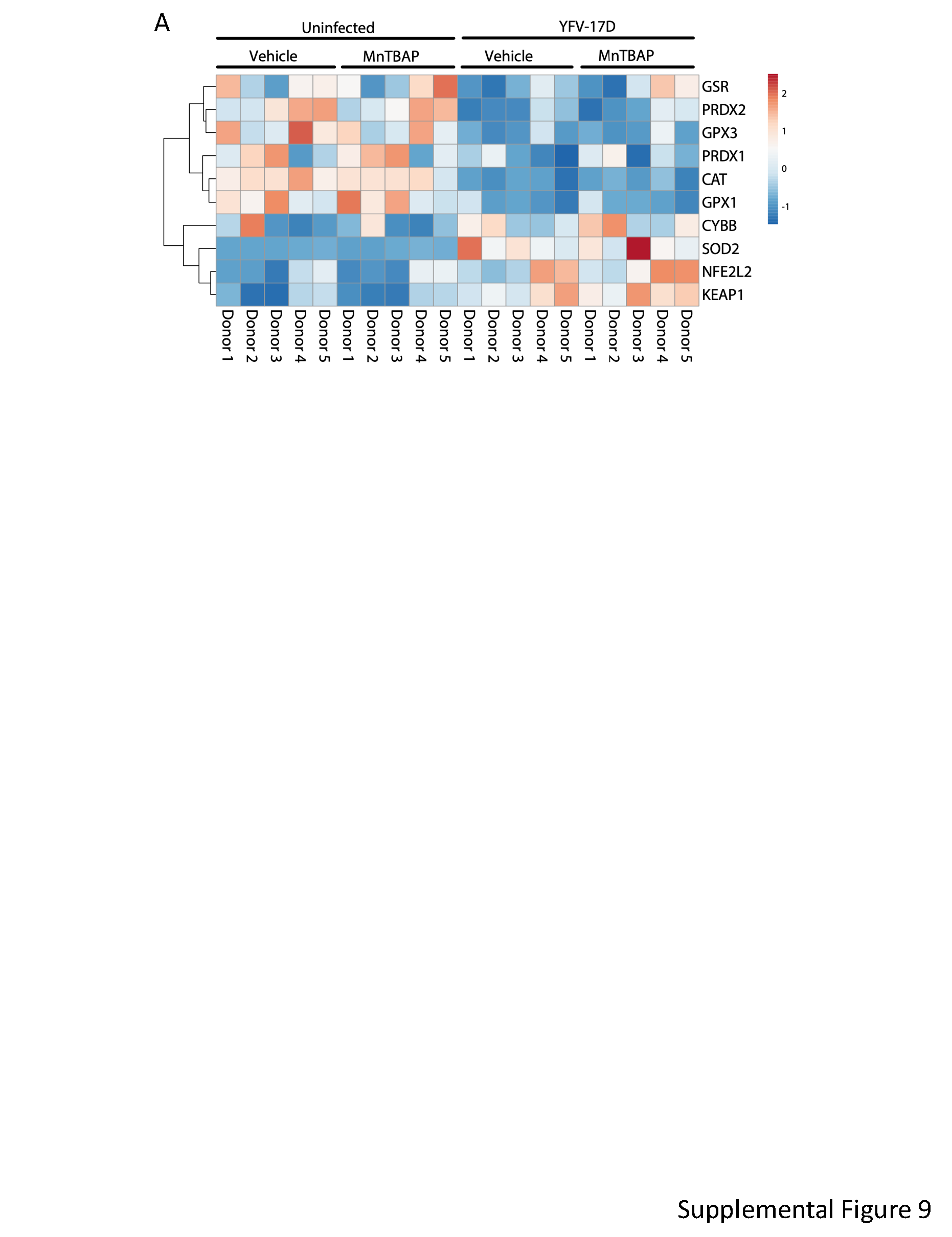

Supplement: S9 Fig — (A) Heatmap comparing the expression of oxidative stress genes in mock and YFV-17D (MOI 0.1) infected human DCs treated with and without MnTBAP. (TIFF) [file ppat.1012561.s009.tiff]
