# Supplementary material for: Mitochondrial hyperactivity and reactive oxygen species drive innate immunity to the yellow fever virus-17D live-attenuated vaccine
Source: PLoS Pathog. 2025 Apr 21;21(4):e1012561. doi: 10.1371/journal.ppat.1012561 (PMC12052391; doi:10.1371/journal.ppat.1012561)
Supplement: S1 Table — (DOCX) [file ppat.1012561.s010.docx]

**S1 Table:** Sequence differences between YFV-17D lab stock and YFV-17D vaccine strain (MT114401.1) molecular clone

| **Genome position of mutation** | **Prevalence** | **Type** | **Nucleotide Change** | **Amino Acid Change** |
| --- | --- | --- | --- | --- |
| 5641 | 99% | Synonymous | A -> G | L1841L |
| 6529 | 99% | Synonymous | C -> T | F2137F |
| 6758 | 100% | Missense | G -> A | V2214I |
| 7319 | 99% | Missense | G -> A | E2401K |
| 9605 | 91% | Missense | G -> A | D3163N |
| 10243 | 100% | Synonymous | A -> G | L3375L |
| 10454 | 99% | Noncoding | G -> A | 3’ UTR |
