# Supplementary material for: Mitochondrial hyperactivity and reactive oxygen species drive innate immunity to the yellow fever virus-17D live-attenuated vaccine
Source: PLoS Pathog. 2025 Apr 21;21(4):e1012561. doi: 10.1371/journal.ppat.1012561 (PMC12052391; doi:10.1371/journal.ppat.1012561)

**S2 Table:** Antiviral immune pathway analysis highlighting DEGs in human monocyte-derived DCs at 48 hpi with YFV-17D and treated with MnTBAP.


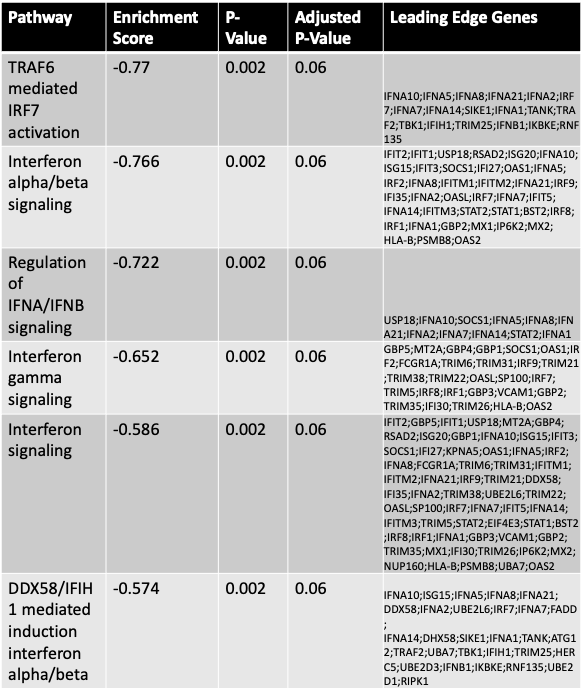

Supplement: S2 Table — (DOCX) [file ppat.1012561.s011.docx]
